# A scoping review of neuromodulation techniques for controlling blood pressure: what are the ups and downs to this approach?

**DOI:** 10.1186/s42234-025-00181-w

**Published:** 2025-08-15

**Authors:** Peter L. Greechan, Ryan G. L. Koh, Paul B. Yoo

**Affiliations:** 1 Institute of Biomedical Engineering, University of Toronto, Toronto, ON M5S 3E2 Canada; 2 KITE Research Institute, Toronto Rehabilitation Institute - University Health Network, Toronto, ON Canada; 3 Edward S. Rogers Sr. Department of Electrical & Computer Engineering Toronto, University of Toronto, Toronto, ON Canada

**Keywords:** Neuromodulation, Blood pressure, Hypertension, Hypotension, Scoping review

## Abstract

**Background and Objectives:**

While there has been rapid progress in research aimed at developing device-based neuromodulation therapies for blood pressure (BP) disorders, there is a paucity of FDA-approved therapies. Currently, the only approved devices for treating resistant hypertension use renal denervation, however, this could soon change as clinical research progresses. With the evolution of interventional strategies for BP regulation, it is important to comprehend the developments to date in order to gauge directions for future research. The objective of this scoping review was to provide the current range of device-based BP neuromodulation approaches, overview salient characteristics of the included studies, address limitations, and detail avenues of further investigation.

**Methods:**

Our review was conducted using the Preferred Reporting Items for Reviews and and Meta-analysis framework. The literature search was performed across the Web of Science Core Collection, Scopus, and Pubmed databases. The search yielded 3503 studies, of which 100 studies remained following the screening process. In the last 10 years, there has been an increase in the number of experimental neurostimulation studies detailing increases and decreases in BP. Of all the included studies, most adopted a non-randomized experimental approach (89%), used animal participants (65%), used invasive neuromodulation methods (74%), and performed acute experiments (84%). More studies documented only depressor responses (49%) compared to pressor responses (35%), and 13% reported both pressor and depressor responses using multiple neural targets.

**Conclusions:**

This review addressed developments in device-based BP neuromodulation, highlighting a dominant focus on treating resistant hypertension compared to hypotensive disorders. While advancements in implantable electrodes have improved spatial selectivity of stimulation, non-invasive neurostimulation devices have become increasingly popular in recent years.

**Supplementary Information:**

The online version contains supplementary material available at 10.1186/s42234-025-00181-w.

## Introduction

Clinical disorders characterized by impaired regulation of arterial blood pressure (BP) can have a wide range of detrimental effects on patient health, resulting in pathological conditions that include chronic elevation (hypertension) or acute drops (hypotension) in arterial BP. Hypertension is the foremost contributor to premature death (responsible for 13.5% of deaths per annum globally), and a prevalent risk factor for cardiovascular disease worldwide (Arima, Barzi and Chalmers 2011). The socioeconomic burden of hypertension is immense, with billions of dollars spent annually in many countries on both direct (medication, hospitalization, etc.) and indirect costs (i.e. out-of-pocket pharmaceutical expenses, absence from work due to illness, etc.) (Wierzejska et al*.* 2020). Its prevalence has been shown to increase with age, afflicting approximately 60% of people in developed countries over the age of 65 (Arima, Barzi and Chalmers 2011; Burnier et al. 2020). Despite the use of at least 3 antihypertensive drugs, there is a subset of patients (10% to 20%) that still exhibit uncontrolled BP and are defined as having resistant hypertension (Sim et al. 2013; Siddiqui and Calhoun 2017; Carey et al. 2019; Leung et al. 2022). Multiple factors can contribute to resistant hypertension, such as an inadequate treatment plan or non-adherence to medication. A rare phenotype of resistant hypertension (called refractory hypertension) is also observed in 0.5% to 5% of patients (Siddiqui and Calhoun 2017), where BP remains uncontrolled despite using at least 5 antihypertensive medications. While the mechanism of this resistance to drug therapy is not fully comprehended, resistant hypertension is associated with comorbidities including chronic kidney disease and obstructive sleep apnea that are linked to elevated sympathetic tone (Hering and Schlaich 2015; Mann 2018).

At the opposite end of the spectrum lies orthostatic hypotension (OH), which is characterized by a sudden and rapid decrease in BP upon standing, making it one of the most common causes of syncope and by association highly correlated with significant morbidity/mortality (Mosnaim et al. 2010; Joseph et al. 2017; Kim 2020). Orthostatic hypotension is defined as a decrease in systolic BP ≥ 20 mmHg or in diastolic BP ≥ 10 mmHg within 3 min of standing (Joseph et al. 2017). Its prevalence varies markedly by age, where approximately 6% of the general population and up to 55% of the elderly population can be affected by OH symptoms (Joseph et al. 2017). Sudden drops in arterial BP pose a significant health threat in the elderly population due to the increased risk of injury and complications from falls (Ungar et al. 2009; Joseph et al. 2017). Syncope and OH in this demographic can have lasting detrimental effects to quality of life and general health. Notably, atrial fibrillation and increased risk of stroke, heart failure, myocardial infarction, and ischemic attacks are all cardiovascular threats associated with hypotension and syncope in the elderly (Rutan et al*.* 1992; Jansen et al. 2015). Difficulty walking as well as psychological stressors including depression and anxiety can manifest in those afflicted by these conditions (Gracie et al. 2006; Jansen et al. 2015). Dizziness, a symptom of OH and often prodromal symptom of syncope, has also been linked to a higher risk of dementia (Ma et al. 2024). Syncope accounts for up to 3% of all emergency department visits (Furlan et al. 2024), and there is a preponderance of young women and elderly persons who experience these syncopal episodes (Ganzeboom et al. 2003; Duncan et al. 2010). An estimated 73% of those who experience vasovagal syncope (temporary loss of consciousness caused by a decline in BP and/or heart rate due to increased vagal tone) are ≥ 60 years of age (Ganzeboom et al. 2003), and female students ≤ 21 years of age reported more syncopal episodes (47%) compared to male students (24%) in a survey conducted at the Amsterdam Academic Medical Centre (Duncan et al. 2010). An estimated 74% of individuals with acute spinal cord injury (SCI) experience OH in the form of dizziness, blurred vision, and syncope (Illman et al. 2000).

Standard first line hypertension treatment entails lifestyle changes, such as reducing sodium intake, moderating alcohol consumption, maintaining a high-fiber, low-fat diet, engaging in regular aerobic exercise, and weight loss (Goit and Yang 2019). Several forms of pharmacotherapy commonly used to treat hypertension are thiazide diuretics, angiotensin-converting enzyme (ACE) inhibitors, α- and β-blockers, and α-adrenoceptor agonists. These medications lower BP by inhibiting sodium reabsorption to reduce fluid volume in the bloodstream and cardiac output, reducing the formation of angiotensin II, blocking adrenergic receptors, and stimulating adrenoceptors in the brain to reduce sympathetic outflow, respectively (Ram 2002). Interestingly, patients often exhibit co-existent hypertension and OH such as in elderly patients taking antihypertensive medications (Cohen et al. 1998; Mosnaim et al. 2010; Zia et al. 2015). Notably, α-blockers and angiotensin II receptor antagonists have been shown to cause OH by reducing vasoconstriction of vessels and systemic vascular tone (Zia et al. 2015). The Antihypertensive and Lipid-Lowering Treatment to Prevent Heart Attack Trial (ALLHAT), however, found that the choice of diuretics, calcium channel blockers, or ACE inhibitor medications did not impact the risk of long-term OH, syncope, or falls in older adults (Juraschek et al. 2019).

Neurodegenerative diseases including Alzheimer’s, Parkinson disease and pure autonomic failure are strongly linked to this comorbidity (Ricci et al. 2015; Joseph et al. 2017; Turana et al. 2022; Pacholko and Iadecola 2024). While it is warranted to consider the potential threat of OH and cardiovascular disease events when treating hypertension, research by Juraschek and colleagues has indicated that a more intense medication-based BP treatment goal (mean BP ≤ 92 mmHg) does not increase the risk of these threats compared to a standard goal (mean BP of 102–107 mmHg) (Juraschek et al. 2018). In this trial, multiple antihypertensive agents were added to the pharmacotherapy regimen as necessary to reach the specified BP target. Among 5 compared trials, a more intensive treatment goal has been associated with a decreased risk of OH (Juraschek et al. 2021). This revelation may impact the risk factors considered when prescribing an antihypertensive medication plan.

It is estimated that up to 25% of hypertensive patients are non-adherent to medications (Tomaszewski et al. 2014), and neurodegenerative disease patients exhibit even lower adherence rates due to cognitive impairment (Shin and Habermann 2016). As an alternative, various modalities of electrical neuromodulation are being developed to control cardiovascular function via the nervous system (Smith et al. 2016). With regards to treating resistant hypertension, renal denervation devices are currently the only therapy to have received Food & Drug Administration (FDA) approval (e.g., ReCor Paradise and Medtronic Symplicity Spyral) (Maini, et al., 2025). Elevated renal sympathetic tone has been linked to the pathogenesis of hypertension by facilitating increased renal vascular resistance, renin activity and sodium retention (DiBona 2002). This elevates sympathetic tone through a cascade of hormonal reactions (known as the renin–angiotensin–aldosterone system) and alters the balance between BP and natriuresis/diuresis (DiBona 2002). Additionally, experimental evidence suggests that the projection of renal afferents to central vasomotor centers upregulates sympathetic outflow (Wyss, Aboukarsh and Oparil 1986; Xu et al. 2015). Thus, the effects of dysfunctional renal nerve activity cause vasoconstriction, increased blood volume and increased cardiac output which can result in hypertension. Renal denervation therapy typically uses radiofrequency or ultrasound emissions via catheter electrodes to ablate the nerves in the renal perivascular space, disrupting the transmission of sympathetic activity to decrease BP (Rey-Garcia and Townsend 2022).

The objective of this scoping review is to highlight the progress made in the field of bioelectronic medicine as a potential intervention for treating hypertensive/hypotensive conditions. The aims of this review are:

- Detail the range of neuromodulation approaches currently being used for controlling hypertension/hypotension.
- Overview the extent to which these approaches demonstrated effectiveness in influencing BP levels.
- Identify gaps in existing literature to inform directions of future research.

## Methods

### Protocol and eligibility criteria

Under the guidelines for Preferred Reporting Items for Systematic Reviews and Meta-analysis (PRISMA) (‘PRISMA Extension for Scoping Reviews (PRISMA-ScR): Checklist and Explanation’ 2018), this scoping review included all studies involving BP control that employed device-based electrical neuromodulation approaches applied to the central or peripheral nervous systems. The selection criteria were (1) original research employing a preclinical or clinical experimental methodology, (2) presented results of neuromodulation on arterial BP, and (3) peer-reviewed article. Publication sources such as conference papers, reviews, theses, editorials and case studies were excluded. Human studies were limited to adult-only participants and pharmacological interventions were omitted.

### Literature search protocol

The search terms used for the initial article selection were (TS = (”blood near/1 press*”) OR TS = (”hyperten*”) OR TS = (”hypoten*”)) AND (TS = (”neuromod*”) OR TS = (”electrical near/1 stim*”) OR TS = (”neurofeedback”) OR TS = (”neural implant*”) OR TS = (”neural near/1 feedback”) OR TS = (”continuous monitor*”)). Searches were carried out across three electronic databases, i.e., Web of Science Core Collection, Scopus, and Pubmed. The search was conducted on Oct. 23, 2024. The search was limited to English language literature. The literature search was limited from Jan. 1, 2000 to the search date, as this date was deemed to represent a sufficiently long period of time to capture all major BP control articles. This choice was supported by the second rapid rise of publications observed in BP in the biomedical domain (Supplementary Fig. 1).

### Selection of sources of evidence

**Fig. 1:**
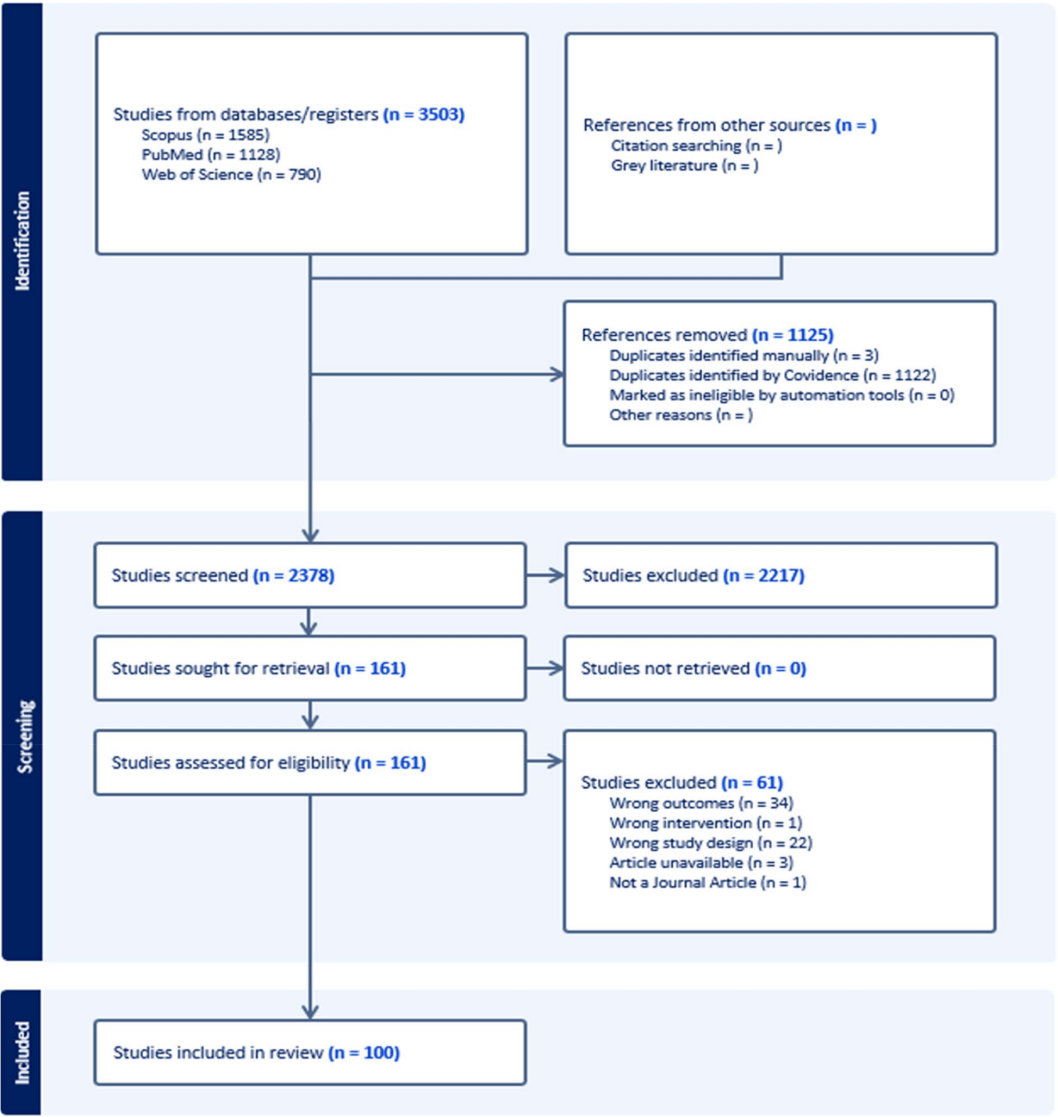
PRISMA flow diagram

The entire screening and extraction process was conducted using Covidence (Veritas Health Innovation, Australia), where an initial review of study titles and abstracts was followed by data extraction according to a customized template (Table 1). Each step of the review process was performed independently by two reviewers and a third reviewer was used to reach a consensus, when necessary. The literature search using the PRISMA framework is summarized in Fig. 1.

**Table 1:** Data extraction template

Category	Characteristic	Description
Publication Details	Title	Title of the publication
	Lead author	First author of the study
	Year	Year of publication
Methodology	Study Design	Type of study conducted (e.g. non-randomized experimental study, randomized controlled trial)
	Participants	Category of subjects used in study (e.g. rodent and small animal, large animal, non-human primate, human)
	Population	Species, if animal participants used, and disease/condition of animal and/or human participants e.g. hypertension)
	Sample size	Number of participants
	Neural interface	Mechanism or method used for neuromodulation
	Neural target	Area of the nervous system targeted for neuromodulation
	Level of intervention	Whether neuromodulation approach was invasive or non-invasive
Outcome	BP modulation direction	Whether neuromodulation increased, decreased, bidirectionally changed, or had no impact on BP

## Results

In the subsections below we present a narrative synthesis of the study characteristics and findings. Note that for some extracted categories, studies could incorporate more than one attribute, which is why the cumulative number of studies for certain characteristics may exceed the total number of 100 studies included in this review. Studies are quantitatively summarized in Supplementary Table 1.

### State of the field

The prominence of preclinical experimental studies (*n* = 65) reflects the emergent nature of the field, where two notable themes were identified: 1) exploratory studies investigating autonomic reflexes characterized by pressor or depressor responses through electrical stimulation, and 2) application of these reflexes for clinical translation. The former group included studies that focused on a mechanistic understanding or characterization of BP reflexes, such as Turner et al. who explored the neural pathway governing baroreflex neuromodulation (Turner et al. 2014). The latter group of studies explored the application of neurostimulation techniques (e.g. stimulation protocol, stimulus modality) for modulating BP. Zhang et al., for instance, investigated the feasibility of eliciting baroreceptor responses by using transcutaneous magnetic stimulation as an alternative to electrical stimulation for targeting the carotid sinus (Zhang et al. 2017). Indeed, various neural targets, including the carotid sinus baroreceptors, have been investigated in clinical studies and represent a shift toward human translation and clinical relevance (Illig et al. 2006). Peer-reviewed publications involving human participants were roughly one third (*n* = 36) of the reviewed studies.

### Relevant clinical indications

BP neuromodulation research placed greater emphasis on treating hypertension than hypotension. This is indicated by the number of studies that reported stimulation-induced depressor responses (*n* = 62 studies); however, pressor-inducing renal nerve stimulation was also used in several studies to determine optimal anatomical ablative sites for antihypertensive renal denervation therapy (Chinushi et al. 2013, 2016, 2020; Hori et al. 2021). Targeting sympathetic renal nerves is difficult given the heterogenous population of fibers among the renal plexus. Thus, this research intended to improve the accuracy of renal denervation by inferring the location of sympatho-excitatory nerve fibers from acute pressor responses. For this reason, the subset of studies that aimed to support neuromodulation-based hypertension research (*n* = 42) is derived from neurostimulation studies that yielded both depressor and pressor BP responses (refer to Supplementary Table 1). In this subset are studies that concentrated on disorders associated with hypertension or specific manifestations of high BP (e.g. autonomic dysreflexia (Sachdeva et al. 2021) and obstructive sleep apnea (Alsharifi et al. 2023)). Among the neural targets used experimentally to reduce BP, there was a noted focus on the carotid sinus baroreceptors (*n* = 9) and the vagus nerve (*n* = 13). This may indicate that FDA-approved device-based therapies for hypertension may expand beyond renal denervation in the near future.

**Fig. 2:**
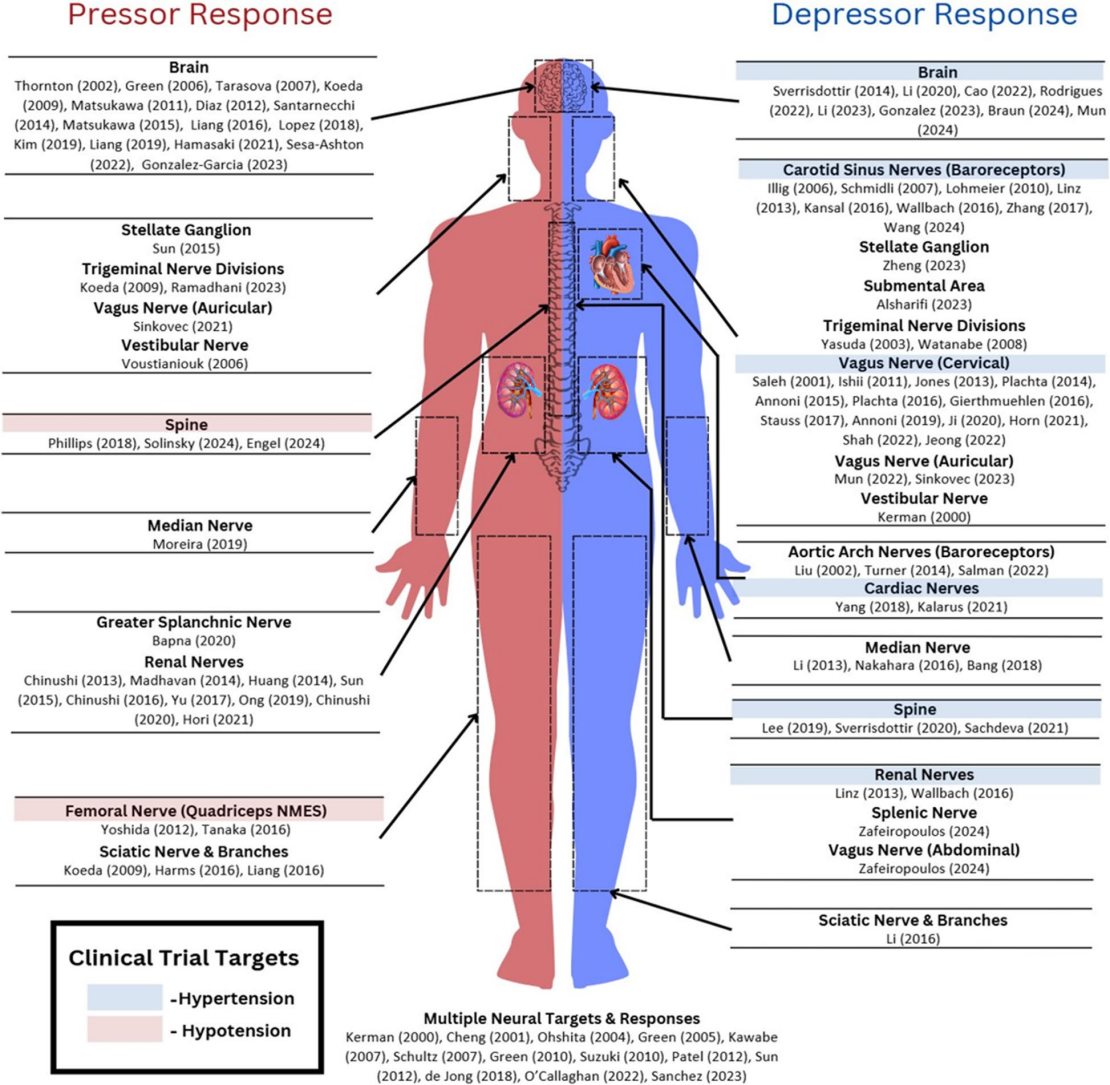
Representation of neuromodulation targets that induced pressor/depressor responses. Highlighted nerve targets represent those associated with clinical trials (available on the ClinicalTrials.gov database) in the recruitment, active, or completed phase, which use neuromodulation to treat hypertension (blue) or hypotension (red)

Only 4 of the 48 studies that documented an evoked pressor reflex had implemented nerve stimulation with the explicit aim of addressing OH (Green et al. 2006; Tarasova et al. 2007; Yoshida et al. 2013; Phillips et al. 2018), while another study intended to treat neurocardiogenic (or vasovagal) syncope (Madhavan et al. 2014). But, as shown in Fig. 2, there were a significant number of studies that observed or demonstrated the feasibility of evoking pressor responses in either preclinical or clinical studies. Some of these studies used nerve stimulation to study neurogenic aspects of hypertension (Kawabe et al. 2007; Liang et al. 2016, 2019; Ong et al. 2019), others focused on the treatment of different forms of cardiovascular dysfunction such as acute myocardial infarction or ventricular arrhythmia (Nakahara et al. 2016; Tanaka et al. 2016; Yu et al. 2017; Moreira et al. 2019; Chinushi et al. 2020; Zheng et al. 2023), while another subset tested novel methods aimed at enhancing nerve stimulation (i.e. selective nerve targeting and non-invasive stimulation) (Antonino et al. 2017; Angius et al. 2018; Horn et al*.* 2021; Jeong et al. 2022).

### Electrical neuromodulation modalities

**Fig. 3:**
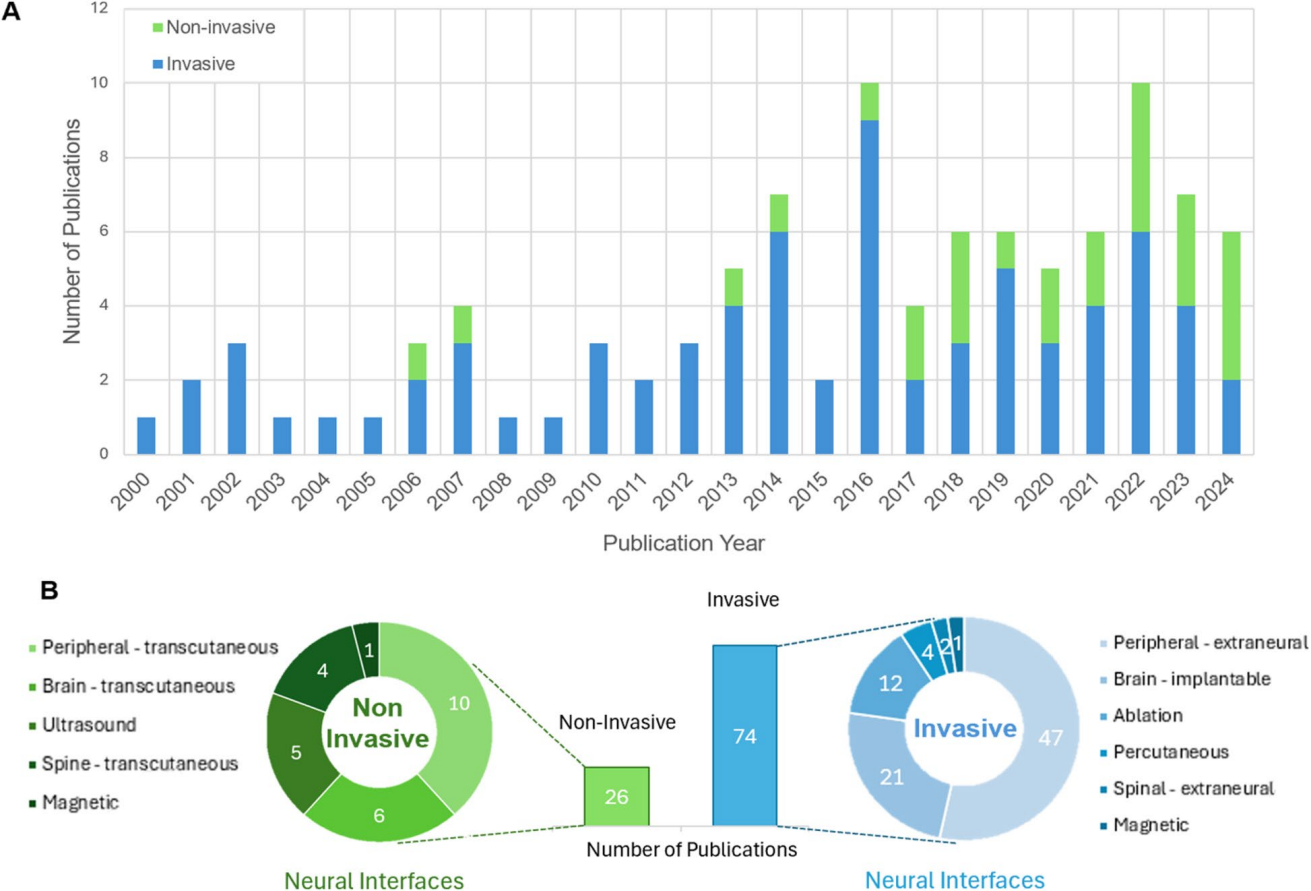
Trend in neural interfaces used for BP neuromodulation. **A** A stacked column chart that illustrates the number of publications using invasive and non-invasive interventional approaches per year. **B** Comparison of the number of publications employing invasive versus non-invasive approaches, along with the distribution of neural interface modalities used to deliver invasive and non-invasive interventions

Development of both implantable neural interfaces and non-invasive devices were prominent trends in the advancement of BP neuromodulation. In this review, instances of invasive modalities being utilized were more frequently observed (*n* = 74) when compared to non-invasive devices (*n* = 26). However, as depicted in Fig. 3, there is an emerging trend in studies investigating non-invasive therapeutic approaches.

Nevertheless, invasive devices are the primary modality for delivering neurostimulation, in part due to their superior selectivity in the stimulation of specific neural targets. This has enabled research to design implantable electrodes that can achieve preferential activation of nerve fibers within a nerve bundle to mitigate off-target effects. To exemplify, studies have explored the concept of spatially selective or fiber-selective activation of cervical vagal afferent fibers to mitigate bradycardic and respiratory side effects, reporting a reduction in these responses while lowering BP (Plachta et al. 2014, 2016; Jeong et al. 2022). Such innovations are possible because implantable electrodes allow for direct contact with the neural target being stimulated.

The main limitation of non-invasive methods is that they are less accurate and precise in stimulating their intended target. More recent studies have focused on non-invasive ultrasound-based neuromodulation as it has a higher spatial resolution compared to non-invasive electrical analogues, attempting to improve stimulation selectivity (Ji et al. 2020; Li et al. 2020, 2023; Cao, et al., 2023). This may contribute to more consistent results in future non-invasive trials, including non-invasive auricular vagus nerve stimulation for antihypertensive therapy. There have been inconsistencies in the reported effectiveness of this technique, which is partially attributed to the difficulty in effectively targeting this stimulus site non-invasively (Šinkovec et al. 2023).

## Discussion

Our scoping review suggests that developments in neuromodulation have preferentially concentrated on the treatment of hypertension compared to hypotension. The key difference being the greater progress made towards clinically translating antihypertensive therapies (Fig. 2). Renal denervation and carotid sinus baroreceptor stimulation appeared most frequently in clinical trials. Previously, the SYMPLICITY HTN-1, HTN-2, and HTN-3 trials conducted using the Symplicity Flex radio-frequency renal ablation device demonstrated mixed efficacy (Symplicity HTN-2 Investigators. 2010; Bhatt et al. 2014; Krum et al. 2014), however, follow-up studies have led to promising results. The SPYRAL HTN-OFF MED trial (ClinicalTrials.gov ID: NCT02439749) and SPYRAL HTN-ON MED trial (NCT02439775) used the Symplicity Spyral device to treat patients with uncontrolled hypertension (office systolic BP ≥ 150 mmHg and < 180 mmHg, diastolic BP ≥ 90 mmHg) after being off medications (SPYRAL HTN-OFF MED) or after receiving medications (SPYRAL HTN-ON MED) (Kandzari et al. 2016). In a 2-year follow-up report, the SPYRAL HTN-ON MED study yielded a significant change in office systolic BP by − 17.4 ± 16.1 mmHg compared to − 9.0 ± 19.4 mmHg in the sham population (*P* = 0.0034) (Kandzari et al. 2025). At the 3 month endpoint, the SPYRAL HTN-OFF MED study yielded a significant decrease in office systolic BP (reduction of 6.6 mmHg, *P* < 0.0001) (Böhm et al. 2020). This study’s short duration was designed to avoid extended medication withdrawal at the cost of being unable to discern long-term effects of off-medication renal denervation. Regardless, both trials demonstrated significant BP changes in their respective cohorts.

Additionally, the RADIANCE trials tested the ultrasound ReCor Paradise ablation system, and the currently active RADIANCE II trial (NCT03614260) reported a significant decrease in BP (−5.4 mmHg systolic BP) at 6 months post-procedure when accounting for titration of antihypertensive medications (Azizi et al. 2024). These 2 devices are FDA-approved for treating resistant hypertension, and the RADIOSOUND-HTN trial (NCT02920034) compared the efficacy of these different ablation systems. In resistant hypertension patients at 6 months after denervation of the main renal arteries, ultrasound ablation produced a significantly greater reduction in ambulatory systolic BP (−12.1 ± 11.5 mmHg) compared to radiofrequency ablation (−6.0 ± 11.0 mm Hg) (Fengler et al. 2023). At 12 months and after optimizing drug treatment, however, this difference was not statistically significant. It was postulated that the greater ablation depth of the ultrasound system or its circumferential catheter design may explain the 6-month difference in BP change compared to that of the spiral-shaped radiofrequency catheter (Fengler et al. 2023).

Renal nerve stimulation has been implemented for improved ablation targeting and has the potential to improve the efficacy of this therapy (Chinushi et al. 2013, 2016, 2020; Hori et al. 2021). Stimulation for enhanced targeting, or renal nerve mapping, has been used to perform effective renal denervation with fewer ablations compared to previous trials. This technique has been implemented in the Sympathetic Mapping/Ablation of Renal Nerves Trial (SMART) (NCT02761811) to treat uncontrolled hypertension patients, resulting in decreased antihypertensive drug dependency and clinically significant reductions in BP among trial participants (Wang et al. 2024).

Furthermore, the Barostim neo device developed for carotid baroreflex activation therapy (BAT) has received FDA approval for treating heart failure (Schäfer et al. 2024), and the Neo Non-Randomized Hypertension Study (NCT01471834) demonstrated sustained acute effectiveness for decreasing BP after a long-term follow up (−14.5 mmHg systolic BP) (Halbach et al. 2015). The recent randomized Nordic BAT pilot trial (NCT02572024) also demonstrated a statistically significant long-term reduction in BP using the Barostim neo, but larger randomized controlled trials are needed to support this device as a treatment for resistant hypertension (Gordin et al. 2024). Wallbach et al. found that this therapy was effective on resistant hypertension patients who had undergone prior renal denervation, indicating that this treatment may also be suitable for non-responders to renal denervation (Wallbach et al. 2016).

Other neuromodulation devices, including cardiac pacemakers, have shown promising results in clinical trials. Kalarus et al. published results from the MODERATO II double-blinded randomized pilot study (NCT02837445), which used the BackBeat Moderato pacemaker device and achieved a net decrease in ambulatory systolic BP of 8.1 mmHg after accounting for control levels (6 months post-implantation) (Kalarus et al. 2021). Vagus nerve stimulation has also been reported to reduce BP (see Fig. 2), however, only limited attempts at clinical translation for hypertension treatment (including a clinical trial case study (NCT00983632)) have been carried out (Rozman et al. 2009). This technique has already been adopted into clinical practice and received FDA approval for epilepsy, depression, obesity, and stroke rehabilitation (Austelle et al*.* 2022; Keser and Feng 2023). Similarly, the eCoin electroacupuncture device, which was used to test median nerve stimulation for lowering BP (NCT02926495), has instead received FDA approval for treating urgency urinary incontinence via tibial nerve stimulation (Lucente et al. 2024).

In contrast to the myriad of hypertension therapies being developed, there are no analogous FDA-approved devices for treating OH; however this does not reflect a lack of patients who might benefit from such therapies. As the population ages, there is a concurrent increase in both hypertensive and hypotensive symptoms, emphasizing the need for developing effective therapeutic techniques to treat both conditions (Gupta and Lipsitz 2007; Abdelhafiz et al. 2018). This need also applies to individuals with chronic SCI in whom autonomic dysfunction can cause significant cardiovascular issues. Depending on the level of spinal injury, either sympathetic, parasympathetic or both sets of autonomic circuits can lose their ability to effectively regulate BP. Failure of these regulatory mechanisms can result in OH and/or autonomic dysreflexia (extremely high BP spikes) (Krassioukov 2009). The primary clinical focus of neuromodulation therapy in this patient population has been to alleviate OH, such as by activating these autonomic circuits or promoting venous return through neuromuscular stimulation (Yoshida et al. 2013, Solinsky et al. 2024). Transcutaneous spinal cord stimulation has been used in clinical trials (NCT05230147, NCT05731986) to induce pressor responses and mitigate or reverse OH symptoms (Phillips et al. 2018; Engel-Haber et al. 2024; Solinsky et al. 2024). Neuromuscular electrical stimulation of the lower limb muscles has also been explored to prevent OH in individuals with SCI (NCT01891110), and although it did not achieve statistically significant results, there was documented improvement in BP stability (Tesini et al*.* 2013). Epidural spinal cord stimulation has also been shown to mitigate the effects of OH in individuals with SCI (Aslan et al*.* 2018), and an active clinical trial (NCT04598035) is investigating this form of stimulation as a means of treating hypertension in neuropathic pain patients. Yoshida et al. used functional electrical stimulation of the same targets of the lower extremities in individuals with SCI to support the therapeutic benefit of this treatment, finding a significant increase in mean BP during applied orthostatic stress (Yoshida et al. 2013).

Technological developments in non-invasive neuromodulation techniques may provide an avenue for accelerating the progress of therapies aimed at treating hypertension and OH. Cao et al. demonstrated a sustained decrease in BP through ultrasound stimulation of the solitary tract nucleus, exemplifying how ultrasound may be used for targeting brain structures (Cao et al. 2023). Non-invasive modalities have become popular in recent years (Fig. 3), and there is potential for ultrasound to expand into other targets within the central nervous systems that have demonstrated pressor responses (*n* = 18, Fig. 2: Brain + Spinal Cord). There is also experimental evidence that deep brain stimulation can increase baroreflex sensitivity and sympathetic outflow (Green et al. 2005, 2006, 2010), suggesting that non-invasive activation of deep brain structures by techniques such as interferential stimulation may be a promising direction for research (Huang et al. 2020).

When targeting cortical regions, transcranial alternating current stimulation may provide an effective means of modulating arterial BP. The application of transcranial alternating current generates an electric field in surrounding cortical regions which causes neural entrainment (synchronizing the brain’s electrical activity with the stimulating current) (Khatoun et al. 2019). This form of stimulation may be a more desirable therapeutic option compared to deep brain stimulation given its non-invasive nature. In Braun and colleagues’ study, this stimulus modality was used to influence activity in the dorsolateral prefrontal cortex, modulating sympathetic outflow to generate transient decreases in BP (Braun et al*.* 2024). A meta-analysis of transcranial magnetic stimulation studies has also found that targeting the same cortical structure with this stimulus modality repeatedly results in decreased BP (Lee et al. 2023). Further exploration of stimulus parameters may allow for these forms of stimulation to increase sympathetic tone and elevate BP for treating hypotensive disorders. Foerster and colleagues, for example, demonstrated both increases and decreases in BP using transcranial magnetic stimulation targeting the primary motor cortex (Foerster et al. 1997). Given that both forms of non-invasive stimulation have shown the ability to elicit BP changes, a direct comparison of the effect each transcranial stimulation modality has on BP and autonomic function should be studied in future research.

The optimization of nerve stimulation paradigms based on observed patterns in neural activity is another area of research with the potential to improve the effectiveness of BP neuromodulation therapy. Autonomic nerve fibers fire with specific bursting sequences and defined frequency bands that often align with cyclic physiological events (e.g. cardiac rhythm) (Malpas 1998). Implementing these waveforms and patterns can augment the response to stimulation, as exemplified by Dibona and Sawin who demonstrated that the use of renal stimulation waveforms resembling in vivo recordings of renal sympathetic bursting activity (diamond-shaped waveforms) enhanced renal vasoconstriction compared to that of conventional (square-shaped) waveforms (DiBona and Sawin 1999). Dibona has also substantiated the frequency dependent response of renal function (i.e. renin secretion rate, tubular sodium reabsorption, and renal blood flow) to renal nerve stimulation (DiBona 2005). The application of such biomimetic stimulation parameters to BP neuromodulation therapy should be further investigated for varying stimulus modalities and anatomical locations.

Because the onset and symptoms of hypotension can occur rapidly, an emphasis should also be placed on developing closed-loop solutions that provide stimulation when BP begins to or is predicted to decline. Advancements in continuous BP monitoring technologies, including photoplethysmography, have the potential to be incorporated as feedback for a closed-loop neuromodulation system to mitigate drops in BP due to hypotension (Park et al. 2022). This concept has been explored in preliminary studies using a system known as the ‘bionic baroreflex’ (Sunagawa and Sugimachi 2010) for combatting both hypertension and hypotension associated with baroreflex failure. This disorder is characterized by impaired carotid sinus baroreceptors, resulting in dysfunctional BP regulation that is difficult to manage with medication and other forms of treatment (Biaggioni et al. 2019). Additionally, Madhavan and colleagues proposed that neurocardiogenic syncope could be treated through renal nerve stimulation (Madhavan et al. 2014). Through the implementation of a closed-loop treatment with feedback to monitor BP and/or heart rate, therapy (e.g. electrical nerve stimulation) may be delivered more effectively by responding to pre-syncopal episodes and preventing unnecessary stimulation when the patient is not experiencing symptoms.

Lastly, a future avenue for BP neuromodulation research is the development of bidirectional strategies. Bidirectional neuromodulation refers to neurostimulation techniques that can selectively increase or decrease BP. Approximately 50% of patients with primary autonomic failure have OH and supine hypertension (Shannon et al. 1997), demonstrating a demographic that could potentially be treated with this therapeutic approach.

### Limitations

The literature cited in this review was limited to publications written in English. Furthermore, we omitted articles that focused on BP monitoring devices because the objective of this review was to assess the current state of functional/clinical effectiveness of neuromodulation approaches.

## Conclusion

In this review, we present an account of original research related to neuromodulation technologies aimed at controlling arterial BP. Our report highlighted the prominence of antihypertensive therapies in the literature, when compared to antihypotensive research. Although the elderly population comprises the majority of hypotensive patients and are at an increased risk of serious injury due to this condition, neuromodulation practices for this cohort remain largely undeveloped. Advancements in device-based neuromodulation approaches may have the potential to help bridge this research gap and further testing is warranted to elucidate the full range of applications for this budding technology. Looking forward, the literature suggests that interventional approaches for BP management will become more efficacious as technology improves, a better understanding of autonomic reflex mechanisms is established, and translational research builds upon current clinical practices.

## Supplementary Information

Supplementary Material 1.

Supplementary Material 2.

## Data Availability

No datasets were generated or analysed during the current study.

## Abbreviations

BP: Blood Pressure
OH: Orthostatic Hypotension
SCI: Spinal Cord Injury
ACE: Angiotensin-Converting Enzyme
ALLHAT: Antihypertensive and Lipid-Lowering Treatment to Prevent Heart Attack Trial
FDA: Food & Drug Administration
PRISMA: Preferred Reporting Items for Systematic Reviews and Meta-analysis
SMART: Sympathetic Mapping/Ablation of Renal Nerves Trial
BAT: Baroreflex Activation Therapy
