# Supplementary material for: A scoping review of neuromodulation techniques for controlling blood pressure: what are the ups and downs to this approach?
Source: Bioelectron Med. 2025 Aug 15;11:19. doi: 10.1186/s42234-025-00181-w (PMC12355777; doi:10.1186/s42234-025-00181-w)
Supplement: Supplementary file 1 — Supplementary Material 1. [file 42234_2025_181_MOESM1_ESM.docx]

**Supplementary Figures & Tables**


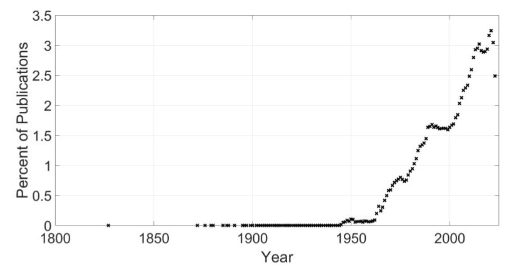


Supplementary Figure 1: Percentage of publications relating to blood pressure indexed by Pubmed since inception to 2023.

SUPPLEMENTARY TABLE 1: Compiled Results of Data Extraction

| Category | | Sub-Category | Number of Citations | Citations |
| --- | --- | --- | --- | --- |
| Study Classification | | Non-Randomized Experimental | 89 | Kerman (2000), Cheng (2001), Saleh (2001), Izumi (2002), Liu (2002), Thornton (2002), Yasuda (2003), Ohshita (2004), Green (2005, 2006, 2010), Illig (2006), Voustianiouk (2006), Tarasova (2007), Schmidli (2007), Kawabe (2007), Watanabe (2008), Koeda (2009), Suzuki (2010), Ishii (2011), Matsukawa (2011), Diaz-Casares (2012), Patel (2012), Sun (2012, 2015), Jones (2013), Li (2013, 2016, 2020, 2023), Linz (2013), Chinushi (2013, 2016, 2020), Huang (2014), Madhavan (2014), Turner (2014), Plachta (2014, 2016), Santarnecchi (2014), Sverrisdóttir (2014), Annoni (2015, 2019), Matsukawa (2015), Gierthmuehlen (2016), Harms (2016), Nakahara (2016), Kansal (2016), Liang (2016, 2019), Zhang (2017), Yu (2017), Phillips (2018), Angius (2018), Bang (2018), de Jong (2018), López-González (2018), Yang (2018), Kim (2019), Lee (2019), Ong (2019), Bapna (2020), Ji (2020), Sverrisdottir (2020), Horn (2021), Hamasaki (2021), Sachdeva (2021), Hori (2021), Cao (2023), Gonzalez-Gonzalez (2022), Jeong (2022), Mun (2022), O’Callaghan (2022), Rodrigues (2022), Salman (2022), Sesa-Ashton (2022), Shah (2022), Alsharifi (2023), Dirr (2023), González-Garc’ia (2023), Zheng (2023), Ramadhani (2023), Sanchez-Larsen (2024), Šinkovec (2023), Braun (2024), Mun (2024), Solinsky (2024), Wang (2024), Zafeiropoulos (2024) |
|  |  | Pilot | 5 | Lohmeier (2010), Yoshida (2012), Stauss (2017), Moreira (2019), Sinkovec (2021) |
|  |  | Cohort | 2 | Wallbach (2016), Kalarus (2021) |
|  |  | Case Series | 2 | Schultz (2007) Engel (2024) |
|  |  | Randomized Controlled | 1 | Antonio (2017) |
|  |  | Quasi-Randomized Controlled | 1 | Tanaka (2016) |
| Research Category | | Clinical | 36 | Thornton (2002), Green (2005, 2006, 2010), Illig (2006), Voustianiouk (2006), Schmidli (2007), Schultz (2007), Pate (2012), Yoshida (2012), Santarnecchi (2014), Sverrisdóttir (2014), Nakahara (2016), Kansal (2016), Tanaka (2016), Wallbach (2016), Antonino (2017), Phillips (2018), Angius (2018), Bang (2018), de Jong (2018), Yang (2018), Moreira (2019), Sverrisdottir (2020), Hamasaki (2021), Kalarus (2021), Sachdeva (2021), Sinkovec (2021), Rodrigues (2022), Sesa-Ashton (2022), Alsharifi (2023), Sanchez-Larsen (2024), Šinkovec (2023), Braun (2024) Engel-Haber (2024), Solinsky (2024) |
|  |  | Preclinical | 65 | Kerman (2000), Cheng (2001), Saleh (2001), Izumi (2002), Liu (2002), Yasuda (2003), Ohshita (2004), Tarasova (2007), Kawabe (2007), Watanabe (2008), Koeda (2009), Lohmeier (2010), Suzuki (2010), Ishii (2011), Matsukawa (2011), Diaz-Casares (2012), Sun (2012, 2015), Jones (2013), Li (2013, 2016, 2020, 2023), Linz (2013), Chinushi (2013, 2016, 2020), Huang (2014), Madhavan (2014), Turner (2014), Plachta (2014, 2016), Annoni (2015, 2019), Matsukawa (2015), Gierthmuehlen (2016), Harms (2016), Liang (2016, 2019), Zhang (2017), Yu (2017), Stauss (2017), López-González (2018), Kim (2019), Lee (2019), Ong (2019), Bapna (2020), Ji (2020), Horn (2021), Sachdeva (2021), Hori (2021), Cao (2023), Gonzalez-Gonzalez (2022), Jeong (2022), Mun (2022), O’Callaghan (2022), Salman (2022), Shah (2022), Dirr (2023), González-Garc’ia (2023), Zheng (2023), Ramadhani (2023), Mun (2024), Wang (2024), Zafeiropoulos (2024) |
| Animal Models | | Rodent & Small Animal | 48 |  |
|  |  | Large Animal | 17 | [28, 32–34, 44, 46, 49, 55, 65, 68–70, 74, 91, 103, 105, 106] |
|  |  | Non-Human Primate | 1 | [68] |
| Acute/Chronic Experimentation | | Acute | 84 | [23, 24, 29, 31–59, 63–94, 96–105, 107–110, 113–115, 118-120, 122] |
|  |  | Chronic/Sub-Chronic | 17 | [25–28, 30, 38, 60–62, 82, 95, 106, 111, 112, 116, 117, 121] |
| Observed response(s) | | Depressor | 49 | [23, 25–27, 29, 30, 37, 38, 45, 47, 48, 50–53, 58–62, 65, 66, 71, 72, 78, 79, 81–84, 87, 89, 90, 94, 95, 98, 100–102, 104–106, 109, 111, 112, 116, 117, 119, 121] |
|  |  | Pressor | 35 | [28, 32–34, 36, 40, 42–44, 46, 56, 57, 63, 64, 67–70, 75, 77, 80, 86, 88, 91, 96, 97, 99, 103, 107, 108, 110, 115, 118, 120, 122] |
|  |  | Depressor & Pressor | 13 | [31, 35, 39, 41, 54, 55, 73, 74, 76, 85, 92, 93, 113] |
|  |  | Statistically Insignificant Change | 3 | [24, 49, 114] |
| Neural Targets | **CNS** | Brain | 32 | [24, 29–31, 36, 39–42, 54, 56, 57, 60, 61, 63, 64, 67, 69, 70, 73, 74, 76, 81, 85, 86, 88, 92, 94, 96, 97, 118, 119] |
|  |  | Spine | 7 | [58, 77, 82, 95, 113, 120, 122] |
|  | **PNS** | Cervical Vagus Nerve | 13 | [25, 26, 37, 45, 48, 50–52, 78, 79, 83, 89, 109] |
|  |  | Renal Nerves | 12 | [32–35, 44, 46, 65, 68, 75, 91, 103, 112] |
|  |  | Carotid Baroreceptors | 9 | [47, 53, 55, 65, 87, 104, 106, 112, 116] |
|  |  | Sciatic Nerve/Sciatic Nerve Branches | 7 | [43, 54, 57, 62, 63, 92, 93] |
|  |  | Aortic Baroreceptors | 6 | [31, 54, 55, 66, 84, 98] |
|  |  | Trigeminal Nerve Divisions | 6 | [49, 57, 74, 80, 100, 102] |
|  |  | Median Nerve | 5 | [27, 55, 59, 72, 107] |
|  |  | Auricular Vagus Nerve | 4 | [71, 90, 108, 114] |
|  |  | Femoral Nerve (Quadriceps NMES) | 2 | [110, 115] |
|  |  | Cardiac Nerves | 2 | [101, 111] |
|  |  | Pulmonary Nerves | 2 | [88, 102] |
|  |  | Vestibular Nerve | 2 | [52, 96] |
|  |  | Submental Area | 1 | [20] |
|  |  | Greater Splanchnic Nerve | 1 | [25] |
| Neural Interface / Nature of Stimulus | Non-Invasive Application | Transcutaneous Electrical Stimulation | 19 | [20, 21, 24, 26, 27, 68, 74, 78, 79, 83, 85, 87, 93, 96, 104, 105, 107, 111, 112] |
|  |  | Ultrasound Stimulation | 3 | [48, 57, 58] |
|  |  | Magnetic Stimulation | 1 | [101] |
|  | Invasive Application | Extraneural Electrical Stimulation | 43 | [22, 23, 25, 28, 29, 31, 32, 34, 35, 40, 42–46, 49–52, 54, 61–63, 65, 71, 72, 75, 76, 80, 81, 84, 86, 88–90, 95, 97–100, 103, 106, 108] |
|  |  | Nerve Ablation | 12 | [29–32, 41, 51, 52, 62, 63, 102, 103, 109] |
|  |  | Percutaneous Stimulation | 4 | [56, 59, 69, 92] |
|  |  | Magnetic Stimulation | 1 | [47] |

SUPPLEMENTARY TABLE 1: Compiled Results of Data Extraction

| Animal Models | | Rodent & Small Animal | 49 | Cheng (2001), Saleh (2001), Liu (2002), Yasuda (2003), Tarasova (2007), Kawabe (2007), Watanabe (2008), Koeda (2009) Suzuki (2010), Ishii, (2011), Matsukawa (2011), Diaz-Casares (2012), Sun (2012), Jones (2013), Li (2013, 2016, 2020, 2023), Turner (2014), Plachta (2014, 2016), Annoni (2015, 2019), Gierthmuehlen (2016), Harms (2016), Liang (2016, 2019), Zhang (2017), Stauss (2017), López-González (2018), Kim (2019), Lee (2019), Ong, (2019), Ji (2020), Horn (2021), Sachdeva (2021), Cao (2023), Gonzalez-Gonzalez (2022), Jeong (2022), Mun (2022), O’Callaghan (2022), Salman (2022), Shah (2022), Dirr (2023), González-Garc’ia (2023), Ramadhani (2023), Mun (2024), Wang (2024), Zafeiropoulos (2024) |
| --- | --- | --- | --- | --- |
|  |  | Large Animal | 17 | Kerman (2000), Izumi (2002), Ohshita (2004), Lohmeier (2010), Matsukawa (2011, 2015), Chinushi (2013, 2016, 2020), Linz (2013), Huang (2014), Madhavan (2014), Sun (2015), Yu (2017), Bapna (2020), Hori (2021), Zheng (2023) |
|  |  | Non-Human Primate | 1 | Madhavan (2014) |
| Acute/Chronic Experimentation | | Acute | 85 | Kerman (2000), Cheng (2001), Saleh (2001), Izumi (2002), Liu (2002), Thornton (2002), Yasuda (2003), Ohshita (2004), Green (2005, 2006, 2010), Illig (2006), Voustianiouk (2006), Kawabe (2007), Schmidli (2007), Schultz (2007), Tarasova (2007), Watanabe (2008), Koeda (2009), Suzuki (2010), Ishii (2011), Matsukawa (2011, 2015), Diaz-Casares (2012), Patel (2012), Sun (2012, 2015), Yoshida (2012), Chinushi (2013, 2016, 2020), Jones (2013), Li (2013), Linz (2013), Huang (2014), Madhavan (2014), Plachta (2014, 2016), Santarnecchi (2014), Sverrisdóttir (2014), Turner (2014), Gierthmuehlen (2016), Harms (2016), Kansal (2016), Liang (2016, 2019), Nakahara (2016), Tanaka (2016), Antonino (2017), Stauss (2017), Yu (2017), Zhang (2017), Angius (2018), de Jong (2018), López-González (2018), Phillips (2018), Yang (2018), Kim (2019), Lee (2019), Moreira (2019), Ong (2019), Ji (2020), Hamasaki (2021), Hori (2021), Horn (2021), Sachdeva (2021), Sinkovec (2021), Gonzalez-Gonzalez (2022), Jeong (2022), Mun (2022), O’Callaghan (2022), Rodrigues (2022), Salman (2022), Sesa-Ashton (2022), Shah (2022), Alsharifi (2023), González-García (2023), Ramadhani (2023), Sanchez-Larsen (2024), Sinkovec (2023), Zheng (2023), Braun (2024), Engel-Haber (2024), Solinsky (2024) |
|  |  | Chronic/Sub-Chronic | 17 | Lohmeier (2010), Annoni (2015, 2019), Li (2016, 2020, 2023), Wallbach (2016), Bang (2018), Bapna (2020), Sverrisdottir (2020), Kalarus (2021), Sachdeva (2021), Cao (2023), Gonzalez-Gonzalez (2022), Dirr (2023), Wang (2024), Zafeiropoulos (2024) |
| Observed response(s) | | Depressor | 49 | Saleh (2001), Liu (2002), Yasuda (2003), Illig (2006), Schmidli (2007), Watanabe (2008), Lohmeier (2010), Ishii (2011), Jones (2013), Li (2013, 2016, 2020, 2023), Linz (2013), Plachta (2014, 2016), Sverrisd´ottir (2014, 2020), Turner (2014), Annoni (2015, 2019), Gierthmuehlen (2016), Kansal (2016), Nakahara (2016), Wallbach (2016), Stauss (2017), Zhang (2017), Bang (2018), Yang (2018), Lee (2019), Ji (2020), Horn (2021), Kalarus (2021), Sachdeva (2021), Cao (2023), Gonzalez-Gonzalez (2022), Jeong (2022), Mun (2022, 2024), Rodrigues (2022), Salman (2022), Shah (2022), Alsharifi (2023), Dirr (2023), Sinkovec (2023), Zheng (2023), Braun (2024), Wang (2024), Zafeiropoulos (2024) |
|  |  | Pressor | 35 | Thornton (2002), Green (2006), Voustianiouk (2006), Tarasova (2007), Koeda (2009), Matsukawa (2011, 2015), Diaz-Casares (2012), Yoshida (2012), Chinushi (2013, 2016, 2020), Huang (2014), Madhavan (2014), Santarnecchi (2014), Sun (2015), Harms (2016), Liang (2016, 2019), Tanaka (2016), Yu (2017), L´opez-Gonz´alez (2018), Phillips (2018), Kim (2019), Moreira (2019), Ong (2019), Bapna (2020), Hamasaki (2021), Hori (2021), Sinkovec (2021), Sesa-Ashton (2022), Gonz´alez-Garc´ıa (2023), Ramadhani (2023), Engel-Haber (2024), Solinsky (2024) |
|  |  | Depressor & Pressor | 13 | [31, 35, 39, 41, 54, 55, 73, 74, 76, 85, 92, 93, 113] |
|  |  | Statistically Insignificant Change | 3 | [24, 49, 114] |
| Neural Targets | **CNS** | Brain | 32 | [24, 29–31, 36, 39–42, 54, 56, 57, 60, 61, 63, 64, 67, 69, 70, 73, 74, 76, 81, 85, 86, 88, 92, 94, 96, 97, 118, 119] |
|  |  | Spine | 7 | [58, 77, 82, 95, 113, 120, 122] |
|  | **PNS** | Cervical Vagus Nerve | 13 | [25, 26, 37, 45, 48, 50–52, 78, 79, 83, 89, 109] |
|  |  | Renal Nerves | 12 | [32–35, 44, 46, 65, 68, 75, 91, 103, 112] |
|  |  | Carotid Baroreceptors | 9 | [47, 53, 55, 65, 87, 104, 106, 112, 116] |
|  |  | Sciatic Nerve/Sciatic Nerve Branches | 7 | [43, 54, 57, 62, 63, 92, 93] |
|  |  | Aortic Baroreceptors | 6 | [31, 54, 55, 66, 84, 98] |
|  |  | Trigeminal Nerve Divisions | 6 | [49, 57, 74, 80, 100, 102] |
|  |  | Median Nerve | 5 | [27, 55, 59, 72, 107] |
|  |  | Auricular Vagus Nerve | 4 | [71, 90, 108, 114] |
|  |  | Femoral Nerve (Quadriceps NMES) | 2 | [110, 115] |
|  |  | Cardiac Nerves | 2 | [101, 111] |
|  |  | Pulmonary Nerves | 2 | [88, 102] |
|  |  | Vestibular Nerve | 2 | [52, 96] |
|  |  | Submental Area | 1 | [20] |
|  |  | Greater Splanchnic Nerve | 1 | [25] |
| Neural Interface / Nature of Stimulus | Non-Invasive Application | Transcutaneous Electrical Stimulation | 19 | [20, 21, 24, 26, 27, 68, 74, 78, 79, 83, 85, 87, 93, 96, 104, 105, 107, 111, 112] |
|  |  | Ultrasound Stimulation | 3 | [48, 57, 58] |
|  |  | Magnetic Stimulation | 1 | [101] |
|  | Invasive Application | Extraneural Electrical Stimulation | 43 | [22, 23, 25, 28, 29, 31, 32, 34, 35, 40, 42–46, 49–52, 54, 61–63, 65, 71, 72, 75, 76, 80, 81, 84, 86, 88–90, 95, 97–100, 103, 106, 108] |
|  |  | Nerve Ablation | 12 | [29–32, 41, 51, 52, 62, 63, 102, 103, 109] |
|  |  | Percutaneous Stimulation | 4 | [56, 59, 69, 92] |
|  |  | Magnetic Stimulation | 1 | [47] |

|  |  | Trigeminal Nerve Divisions | 6 | [49, 57, 74, 80, 100, 102] |
| --- | --- | --- | --- | --- |
|  |  | Median Nerve | 5 | [27, 55, 59, 72, 107] |
|  |  | Auricular Vagus Nerve | 4 | [71, 90, 108, 114] |
|  |  | Femoral Nerve (Quadriceps NMES) | 2 | [110, 115] |
|  |  | Cardiac Nerves | 2 | [101, 111] |
|  |  | Stellate Ganglion | 2 | [91, 105] |
|  |  | Vestibular Nerve | 2 | [55, 99] |
|  |  | Submental Area | 1 | [23] |
|  |  | Greater Splanchnic Nerve | 1 | [28] |
|  |  | Splenic Nerve | 1 | [117] |
|  |  | Abdominal Vagus Nerve | 1 | [121] |
| Neural Interface / Nature of Stimulus | Non-Invasive Application | Transcutaneous Electrical Stimulation | 21 | [23, 24, 27, 29, 30, 71, 77, 81, 82, 86, 88, 90, 96, 99, 107, 108, 110, 114, 115, 120, 122] |
|  |  | Ultrasound Stimulation | 4 | [51, 60, 61, 117] |
|  |  | Magnetic Stimulation | 1 | [104] |
|  | Invasive Application | Peripheral Extraneural Electrical Stimulation | 47 | [25, 26, 28, 31, 32, 34, 35, 37, 38, 43, 45–49, 52– 55, 57, 64–66, 68, 74, 75, 78, 79, 83, 84, 87, 89, 91–93, 98, 100–103, 106, 109, 111, 116, 118, 119, 121] |
|  |  | Brain Stimulation | 21 | [28, 33, 36–39, 51, 53, 54, 60, 61, 64, 66, 67, 70, 73, 77, 82, 89, 91, 94] |
|  |  | Nerve Ablation | 12 | [32–35, 44, 54, 55, 65, 66, 105, 106, 112] |
|  |  | Percutaneous Stimulation | 4 | [59, 62, 72, 95] |
|  |  | Spinal Nerve Stimulation | 2 | [58, 113] |
|  |  | Magnetic Stimulation | 1 | [50] |

SUPPLEMENTARY TABLE 1: Compiled Results of Data Extraction

| Observed response(s) | | Pressor | 35 | Hamasaki (2021), Hori (2021), Sinkovec (2021), Sesa-Ashton (2022), Gonz´alez-Garc´ıa (2023), Ramadhani (2023), Engel-Haber (2024), Solinsky (2024) |
| --- | --- | --- | --- | --- |
|  |  | Depressor & Pressor | 13 | Kerman (2000), Cheng (2001), Ohshita (2004), Green (2005, 2010), Kawabe (2007), Schultz (2007), Suzuki (2010), Patel (2012), Sun (2012), de Jong (2018), O’Callaghan (2022), Sanchez-Larsen (2024) |
|  |  | Statistically Insignificant Change | 3  P | Izumi (2002), Antonino (2017), Angius (2018)  32 |
| Neural Targets | **CNS** | Brain | 32 | Cheng (2001), Thornton (2002), Ohshita (2004), Green (2005, 2006, 2010), Kawabe (2007), Tarasova (2007), Koeda (2009), Matsukawa (2011, 2015), Diaz-Casares (2012), Patel (2012), Sun (2012), Santarnecchi (2014), Sverrisd´ottir (2014), Liang (2016, 2019), Angius (2018), L´opez-Gonz´alez (2018), Kim (2019), Li (2020), Hamasaki (2021), Cao (2023), O’Callaghan (2022), Rodrigues (2022), Sesa-Ashton (2022), Gonz´alez-Garc´ıa (2023), Sanchez-Larsen (2024), Braun (2024), Mun (2024) |
|  |  | Spine | 7 | Schultz (2007), Phillips (2018), Lee (2019), Sverrisdotir (2020), Sachdeva (2021), Engel-Haber (2024), Solinsky (2024) |
|  | **PNS** | Cervical Vagus Nerve | 13 | Saleh (2001), Ishii (2011), Jones (2013), Plachta (2014, 2016), Annoni (2015, 2019), Gierthmuehlen (2016), Stauss (2017), Ji (2020), Horn (2021), Jeong (2022), Shah (2022) |
|  |  | Renal Nerves | 12 | Chinushi (2013, 2016, 2020), Linz (2013), Huang (2014), Madhavan (2014), Sun (2015), Wallbach (2016), Yu (2017), de Jong (2018), Ong (2019), Hori (2021) |
|  |  | Carotid Baroreceptors | 9 | Kerman (2000), Illig (2006), Schmidli (2007), Lohmeier (2010), Linz (2013), Kansal (2016), Wallbach (2016), Zhang (2017), Wang (2024) |
|  |  | Sciatic Nerve/Sciatic Nerve Branches | 7 | Kawabe (2007), Koeda (2009), Suzuki (2010), Sun (2012), Harms (2016), Li (2016), Liang (2016) |
|  |  | Aortic Baroreceptors | 6 | Kerman (2000), Cheng (2001), Liu (2002), Kawabe (2007), Turner (2014), Salman (2022) |
|  |  | Trigeminal Nerve Divisions | 6 | Izumi (2002), Yasuda (2003), Ohshita (2004), Watanabe (2008), Koeda (2009), Ramadhani (2023) |
|  |  | Median Nerve | 5 | Kerman (2000), Li (2013), Nakahara (2016), Bang (2018), Moreira (2019) |
|  |  | Auricular Vagus Nerve | 4 | Antonino (2017), Sinkovec (2021, 2023), Mun (2022) |
|  |  | Femoral Nerve (Quadriceps NMES) | 2 | Yoshida (2012), Tanaka (2016) |
|  |  | Pulmonary Nerves | 2 | [49, 57, 74, 80, 100, 102] |
|  |  | Vestibular Nerve | 2 | [27, 55, 59, 72, 107] |
|  |  | Submental Area | 1 | [71, 90, 108, 114] |
|  |  | Greater Splanchnic Nerve | 1 | [110, 115] |
|  |  | Transcutaneous Electrical Stimulation | 19 | [101, 111] |
|  |  | Ultrasound Stimulation | 3 | [88, 102] |
|  |  | Magnetic Stimulation | 1 | [52, 96] |
|  |  | Extraneural Electrical Stimulation | 43 | [20] |
|  |  | Nerve Ablation | 12 | [25] |
| Neural Interface / Nature of Stimulus | Non-Invasive Application | Percutaneous Stimulation | 4 | [20, 21, 24, 26, 27, 68, 74, 78, 79, 83, 85, 87, 93, 96, 104, 105, 107, 111, 112] |
|  |  | Magnetic Stimulation | 1 | [48, 57, 58] |
|  |  |  |  | [101] |
|  |  |  |  |  |
|  |  |  |  |  |
|  |  |  |  |  |
|  |  |  |  |  |

SUPPLEMENTARY TABLE 1

| Category | | Sub-Category | Number of Citations | Citations |
| --- | --- | --- | --- | --- |
| Study Classification | | Non-Randomized Experimental | 89 | Kerman (2000), Cheng (2001), Saleh (2001), Izumi (2002), Liu (2002), Thornton (2002), Yasuda (2003), Ohshita (2004), Green (2005, 2006, 2010), Illig (2006), Voustianiouk (2006), Tarasova (2007), Schmidli (2007), Kawabe (2007), Watanabe (2008), Koeda (2009), Suzuki (2010), Ishii (2011), Matsukawa (2011), Diaz-Casares (2012), Patel (2012), Sun (2012, 2015), Jones (2013), Li (2013, 2016, 2020, 2023), Linz (2013), Chinushi (2013, 2016, 2020), Huang (2014), Madhavan (2014), Turner (2014), Plachta (2014, 2016), Santarnecchi (2014), Sverrisdóttir (2014), Annoni (2015, 2019), Matsukawa (2015), Gierthmuehlen (2016), Harms (2016), Nakahara (2016), Kansal (2016), Liang (2016, 2019), Zhang (2017), Yu (2017), Phillips (2018), Angius (2018), Bang (2018), de Jong (2018), López-González (2018), Yang (2018), Kim (2019), Lee (2019), Ong (2019), Bapna (2020), Ji (2020), Sverrisdottir (2020), Horn (2021), Hamasaki (2021), Sachdeva (2021), Hori (2021), Cao (2022), Gonzalez-Gonzalez (2022), Jeong (2022), Mun (2022), O’Callaghan (2022), Rodrigues (2022), Salman (2022), Sesa-Ashton (2022), Shah (2022), Alsharifi (2023), Dirr (2023), González-Garc’ia (2023), Zheng (2023), Ramadhani (2023), Sanchez-Larsen (2023), Šinkovec (2023), Braun (2024), Mun (2024), Solinsky (2024), Wang (2024), Zafeiropoulos (2024) |
|  |  | Pilot | 5 | Lohmeier (2010), Yoshida (2012), Stauss (2017), Moreira (2019), Sinkovec (2021) |
|  |  | Cohort | 2 | Wallbach (2016), Kalarus (2021) |
|  |  | Case Series | 2 | Schultz (2007) Engel (2024) |
|  |  | Randomized Controlled | 1 | Antonio (2017) |
|  |  | Quasi-Randomized Controlled | 1 | Tanaka (2016) |
| Research Category | | Clinical | 36 | Thornton (2002), Green (2005, 2006, 2010), Illig (2006), Voustianiouk (2006), Schmidli (2007), Schultz (2007), Pate (2012), Yoshida (2012), Santarnecchi (2014), Sverrisdóttir (2014), Nakahara (2016), Kansal (2016), Tanaka (2016), Wallbach (2016), Antonino (2017), Phillips (2018), Angius (2018), Bang (2018), de Jong (2018), Yang (2018), Moreira (2019), Sverrisdottir (2020), Hamasaki (2021), Kalarus (2021), Sachdeva (2021), Sinkovec (2021), Rodrigues (2022), Sesa-Ashton (2022), Alsharifi (2023), Sanchez-Larsen (2023), Šinkovec (2023), Braun (2024) Engel-Haber (2024), Solinsky (2024) |
|  |  | Preclinical | 65 | [25, 26, 28, 30–34, 36–38, 43–46, 48–52, 54–71, 73–75, 78–80, 82–84, 89, 91–93, 96, 98, 100, 102–106, 109 116-119, 121] |
| Animal Models | | Rodent & Small Animal | 48 | [25, 26, 30, 31, 36–38, 43, 45, 48, 50–52, 54, 56–64, 66, 67, 70, 71, 73, 75, 78–80, 82–84, 89, 92, 93, 96, 98, 100, 102, 104, 109] |
|  |  | Large Animal | 17 | [28, 32–34, 44, 46, 49, 55, 65, 68–70, 74, 91, 103, 105, 106] |
|  |  | Non-Human Primate | 1 | [68] |
| Acute/Chronic Experimentation | | Acute | 84 | [23, 24, 29, 31–59, 63–94, 96–105, 107–110, 113–115, 118-120, 122] |
|  |  | Chronic/Sub-Chronic | 17 | [25–28, 30, 38, 60–62, 82, 95, 106, 111, 112, 116, 117, 121] |
| Observed response(s) | | Depressor | 49 | [23, 25–27, 29, 30, 37, 38, 45, 47, 48, 50–53, 58–62, 65, 66, 71, 72, 78, 79, 81–84, 87, 89, 90, 94, 95, 98, 100–102, 104–106, 109, 111, 112, 116, 117, 119, 121] |
|  |  | Pressor | 35 | [28, 32–34, 36, 40, 42–44, 46, 56, 57, 63, 64, 67–70, 75, 77, 80, 86, 88, 91, 96, 97, 99, 103, 107, 108, 110, 115, 118, 120, 122] |
|  |  | Depressor & Pressor | 13 | [31, 35, 39, 41, 54, 55, 73, 74, 76, 85, 92, 93, 113] |
|  |  | Statistically Insignificant Change | 3 | [24, 49, 114] |
| Neural Targets | **CNS** | Brain | 32 | [24, 29–31, 36, 39–42, 54, 56, 57, 60, 61, 63, 64, 67, 69, 70, 73, 74, 76, 81, 85, 86, 88, 92, 94, 96, 97, 118, 119] |
|  |  | Spine | 7 | [58, 77, 82, 95, 113, 120, 122] |
|  | **PNS** | Cervical Vagus Nerve | 13 | [25, 26, 37, 45, 48, 50–52, 78, 79, 83, 89, 109] |
|  |  | Renal Nerves | 12 | [32–35, 44, 46, 65, 68, 75, 91, 103, 112] |
|  |  | Carotid Baroreceptors | 9 | [47, 53, 55, 65, 87, 104, 106, 112, 116] |
|  |  | Sciatic Nerve/Sciatic Nerve Branches | 7 | [43, 54, 57, 62, 63, 92, 93] |
|  |  | Aortic Baroreceptors | 6 | [31, 54, 55, 66, 84, 98] |
|  |  | Trigeminal Nerve Divisions | 6 | [49, 57, 74, 80, 100, 102] |
|  |  | Median Nerve | 5 | [27, 55, 59, 72, 107] |
|  |  | Auricular Vagus Nerve | 4 | [71, 90, 108, 114] |
|  |  | Femoral Nerve (Quadriceps NMES) | 2 | [110, 115] |
|  |  | Cardiac Nerves | 2 | [101, 111] |
|  |  | Pulmonary Nerves | 2 | [88, 102] |
|  |  | Vestibular Nerve | 2 | [52, 96] |
|  |  | Submental Area | 1 | [20] |
|  |  | Greater Splanchnic Nerve | 1 | [25] |
| Neural Interface / Nature of Stimulus | Non-Invasive Application | Transcutaneous Electrical Stimulation | 19 | [20, 21, 24, 26, 27, 68, 74, 78, 79, 83, 85, 87, 93, 96, 104, 105, 107, 111, 112] |
|  |  | Ultrasound Stimulation | 3 | [48, 57, 58] |
|  |  | Magnetic Stimulation | 1 | [101] |
|  | Invasive Application | Extraneural Electrical Stimulation | 43 | [22, 23, 25, 28, 29, 31, 32, 34, 35, 40, 42–46, 49–52, 54, 61–63, 65, 71, 72, 75, 76, 80, 81, 84, 86, 88–90, 95, 97–100, 103, 106, 108] |
|  |  | Nerve Ablation | 12 | [29–32, 41, 51, 52, 62, 63, 102, 103, 109] |
|  |  | Percutaneous Stimulation | 4 | [56, 59, 69, 92] |
|  |  | Magnetic Stimulation | 1 | [47] |

SUPPLEMENTARY TABLE 1: Compiled Results of Data Extraction

| Neural Targets | PNS | Cardiac Nerves | 2 | Yang (2018), Kalarus (2021) |
| --- | --- | --- | --- | --- |
|  |  | Stellate Ganglion | 2 | Sun (2015), Zheng (2023) |
|  |  | Vestibular Nerve | 1 | Kerman (2000), Voustianiouk (2006) |
|  |  | Submental Area | 1 | Alsharifi (2023) |
|  |  | Greater Splanchnic Nerve | 1 | Bapna (2020) |
|  |  | Splenic Nerve | 1 | Zafeiropoulos (2024) |
|  |  | Abdominal Vagus Nerve | 1 | Dirr (2023) |
| Neural Interface / Nature of Stimulus | **Non-Invasive Application** | Transcutaneous Electrical Stimulation | 21 | Voustianiouk (2006), Tarasova (2007), Yoshida (2012), Santarnecchi (2014), Tanaka (2016), Antonino (2017), Angius (2018), Bang (2018), Phillips (2018), Moreira (2019), Sachdeva (2021), Sinkovec (2021, 2023), Cao (2023), Mun (2022), Rodrigues (2022), Sesa-Ashton (2022), Alsharifi (2023), Braun (2024), Engel-Haber (2024), Solinsky (2024) |
|  |  | Ultrasound | 4 | Ji (2020), Li (2020, 2023), Zafeiropoulos (2024) |
|  |  | Magnetic Stimulation | 1 | Zhang (2017) |
|  | **Invasive Application** | Peripheral Extraneural Electrical Stimulation | 48 | Kerman (2000), Cheng (2001), Saleh (2001), Izumi (2002), Liu (2002), Yasuda (2003), Ohshita (2004), Illig (2006), Kawabe (2007), Schmidli (2007), Watanabe (2008), Koeda (2009), Lohmeier (2010), Suzuki (2010), Ishii (2011), Sun (2012, 2015), Chinushi (2013, 2020), Jones (2013), Linz (2013), Huang (2014), Madhavan (2014), Plachta (2014, 2016), Turner (2014), Annoni (2015, 2019), Gierthmuehlen (2016), Harms (2016), Kansal (2016), Stauss (2017), Yu (2017), de Jong (2018), Yang (2018), Liang (2019), Ong (2019), Bapna (2020), Horn (2021), Kalarus (2021), Gonzalez-Gonzalez (2022), Salman (2022), Shah (2022), Dirr (2023), Gonz´alez-Garc´ıa (2023), Ramadhani (2023), Mun (2024), Wang (2024) |
|  |  | Brain Stimulation | 20 | Cheng (2001), Thornton (2002), Green (2005, 2006, 2010), Kawabe (2007), Koeda (2009), Matsukawa (2011, 2015), Diaz-Casares (2012), Patel (2012), Sun (2012), Sverrisd´ottir (2014), Liang (2016, 2019), L´opez-Gonz´alez (2018), Kim (2019), Hamasaki (2021), O’Callaghan (2022), Sanchez-Larsen (2024) |
|  |  | Nerve Ablation | 12 | Nerve Ablation |
|  |  | Percutaneous Stimulation | 4 | Percutaneous Stimulation |

SUPPLEMENTARY TABLE 1: Compiled Results of Data Extraction

| Neural Interface / Nature of Stimulus | Invasive Application | Nerve Ablation | 12 | Kerman (2000), Liu (2002), Kawabe (2007), Lohmeier (2010), Chinushi (2013, 2016, 2020), Linz (2013), Wallbach (2016), de Jong (2018), Hori (2021), Zheng (2023) |
| --- | --- | --- | --- | --- |
|  |  | Percutaneous Stimulation | 4 | Li (2013, 2016), Nakahara (2016), Sverrisdottir (2020) |
|  |  | Spinal Cord Stimulation | 2 | Schultz (2007), Lee (2019) |
|  |  | Magnetic Stimulation | 1 | Jeong (2022) |
