# Supplementary material for: A scoping review of neuromodulation techniques for controlling blood pressure: what are the ups and downs to this approach?
Source: Bioelectron Med. 2025 Aug 15;11:19. doi: 10.1186/s42234-025-00181-w (PMC12355777; doi:10.1186/s42234-025-00181-w)
Supplement: Supplementary file 2 — Supplementary Material 2. [file 42234_2025_181_MOESM2_ESM.docx]

**Supplementary Table 1 References**

Alsharifi A, Kaltsakas G, Pengo MF, Parati G, Serna-Pascual M, Rafferty G, et al. The effect of

transcutaneous electrical stimulation of the submental area on the cardiorespiratory response in normal and awake subjects. Front Physiol. 2023;14:1089837.

Angius L, Marcora SM, Hopker JG, Mauger AR. The effect of anodal transcranial direct current

stimulation over left and right temporal cortex on the cardiovascular response: a comparative study. Front Physiol. 2018;9:430134.

Annoni EM, Van Helden D, Guo Y, Levac B, Libbus I, KenKnight BH, et al. Chronic low-level vagus

nerve stimulation improves long-term survival in salt-sensitive hypertensive rats. Front Physiol. 2019;10:25.

Annoni EM, Xie X, Lee SW, Libbus I, KenKnight BH, Osborn JW, et al. Intermittent electrical

stimulation of the right cervical vagus nerve in salt-sensitive hypertensive rats: effects on blood pressure, arrhythmias, and ventricular electrophysiology. Physiol Rep. 2015;3(8):e12476.

Antonino D, Teixeira AL, Maia-Lopes PM, Souza MC, Sabino-Carvalho JL, Murray AR, et al. Non-

invasive vagus nerve stimulation acutely improves spontaneous cardiac baroreflex sensitivity in healthy young men: A randomized placebo-controlled trial. Brain Stimul. 2017;10(5):875–81.

Bang SK, Ryu Y, Chang S, Im CK, Bae JH, Gwak YS, et al. Attenuation of hypertension by C-fiber

stimulation of the human median nerve and the concept-based novel device. Sci Rep. 2018;8(1):14967.

Bapna A, Adin C, Engelman ZJ, Fudim M. Increasing blood pressure by greater splanchnic nerve

stimulation: a feasibility study. J Cardiovasc Transl Res. 2020;13:509–18.

Braun JA, Patel M, Henderson LA, Dawood T, Macefield VG. Electrical stimulation of the ventromedial

prefrontal cortex modulates muscle sympathetic nerve activity and blood pressure. Cerebral Cortex. 2024;34(1):bhad422.

Cao F, Zhang J, Li D, Wang M, Lai C, Xu T, et al. Non-invasive ultrasound modulation of solitary tract

nucleus exerts a sustainable antihypertensive effect in spontaneously hypertensive rats. IEEE Trans Biomed Eng. 2023;70(6):1869-78.

Cheng Z Bin, Kobayashi M, Nosaka S. Effects of optic tract stimulation on baroreflex vagal bradycardia

in rats. Clin Exp Pharmacol Physiol. 2001;28(9):721–8.

Chinushi M, Izumi D, Iijima K, Suzuki K, Furushima H, Saitoh O, et al. Blood pressure and autonomic

responses to electrical stimulation of the renal arterial nerves before and after ablation of the renal artery. Hypertension. 2013;61(2):450–6.

Chinushi M, Saitoh O, Sugai A, Oikawa A, Watanabe J, Furushima H. Enhanced arrhythmogenic

potential induced by renal autonomic nerve stimulation: Role of renal artery catheter ablation. Heart Rhythm. 2020;17(1):133–41.

Chinushi M, Suzuki K, Saitoh O, Furushima H, Iijima K, Izumi D, et al. Electrical stimulation–based

evaluation for functional modification of renal autonomic nerve activities induced by catheter ablation. Heart Rhythm. 2016;13(8):1707–15.

de Jong MR, Hoogerwaard AF, Adiyaman A, Smit JJJ, Heeg JE, van Hasselt BAAM, et al. Renal nerve

stimulation identifies aorticorenal innervation and prevents inadvertent ablation of vagal nerves during renal denervation. Blood Press. 2018;27(5):271–9.

Diaz-Casares A, López-González MV, Peinado-Aragonés CA, González-Barón S, Dawid-Milner MS.

Parabrachial complex glutamate receptors modulate the cardiorespiratory response evoked from hypothalamic defense area. Autonomic Neuroscience. 2012;169(2):124–34.

Dirr EW, Jiracek LG, Baekey DM, J. Martyniuk C, Otto KJ, Zubcevic J. Subdiaphragmatic vagal nerve

stimulation attenuates the development of hypertension and alters nucleus of the solitary tract transcriptional networks in the spontaneously hypertensive rat. Physiol Genomics. 2023;55(12):606–17.

Engel-Haber E, Bheemreddy A, Bayram MB, Ravi M, Zhang F, Su H, et al. Neuromodulation in Spinal

Cord Injury Using Transcutaneous Spinal Stimulation—Mapping for a Blood Pressure Response: A Case Series. Neurotrauma Rep. 2024;5(1):845–56.

Gierthmuehlen M, Plachta DTT. Effect of selective vagal nerve stimulation on blood pressure, heart rate

and respiratory rate in rats under metoprolol medication. Hypertension Research. 2016;39(2):79–87.

González-Garcia M, Carrillo-Franco L, Peinado-Aragonés CA, et al. Impact of the glutamatergic

neurotransmission within the A5 region on the cardiorespiratory response evoked from the midbrain dlPAG. Pflügers Archiv-European Journal of Physiology. 2023;475(4):505–16.

Gonzalez-Gonzalez MA, Romero K, Beitter J, Lloyd D, Lam D V, Hernandez-Reynoso AG, et al. Renal

nerve activity and arterial depressor responses induced by neuromodulation of the deep peroneal nerve in spontaneously hypertensive rats. Front Neurosci. 2022;16:726467.

Green AL, Hyam JA, Williams C, Wang S, Shlugman D, Stein JF, et al. Intra-operative deep brain

stimulation of the periaqueductal grey matter modulates blood pressure and heart rate variability in humans. Neuromodulation: Technology at the Neural Interface. 2010;13(3):174–81.

Green AL, Wang S, Owen SLF, Paterson DJ, Stein JF, Aziz TZ. Controlling the heart via the brain: a

potential new therapy for orthostatic hypotension. Neurosurgery. 2006;58(6):1176–83.

Green AL, Wang S, Owen SLF, Xie K, Liu X, Paterson DJ, et al. Deep brain stimulation can regulate

arterial blood pressure in awake humans. Neuroreport. 2005;16(16):1741–5.

Hamasaki T, Yamakawa T, Fujiwara K, Harashima H, Nakamura K, Ikuta Y, et al. Sympathetic

hyperactivity, hypertension, and tachycardia induced by stimulation of the ponto-medullary junction in humans. Clinical Neurophysiology. 2021;132(6):1264–73.

Harms JE, Copp SW, Kaufman MP. Low-frequency stimulation of group III and IV hind limb afferents

evokes reflex pressor responses in decerebrate rats. Physiol Rep. 2016;4(20):e13001.

Hori Y, Temma T, Wooten C, Sobowale C, Tahmasian S, Chan C, et al. Aorticorenal ganglion as a novel

target for renal neuromodulation. Heart Rhythm. 2021;18(10):1745–57.

Horn CC, Forssell M, Sciullo M, Harms JE, Fulton S, Mou C, et al. Hydrogel-based electrodes for

selective cervical vagus nerve stimulation. J Neural Eng. 2021;18(5):055008.

Huang B, Yu L, Scherlag BJ, Wang S, He BO, Yang K, et al. Left renal nerves stimulation facilitates

ischemia-induced ventricular arrhythmia by increasing nerve activity of left stellate ganglion. J Cardiovasc Electrophysiol. 2014;25(11):1249–56.

Illig KA, Levy M, Sanchez L, Trachiotis GD, Shanley C, Irwin E, et al. An implantable carotid sinus

stimulator for drug-resistant hypertension: surgical technique and short-term outcome from the multicenter phase II Rheos feasibility trial. J Vasc Surg. 2006;44(6):1213–8.

Ishii H, Niioka T, Izumi H. Parasympathetic reflex vasodilatation in the masseter muscle compensates

for carotid hypoperfusion during the vagus-mediated depressor response. Brain Res. 2011;1370:145–53.

Izumi H, Mizuta K, Kuchiiwa S. Simultaneous measurement of parasympathetic reflex vasodilator and

arterial blood pressure responses in the cat. Brain Res. 2002;952(1):61–70.

Jeong H, Cho A, Ay I, Bonmassar G. Short-pulsed micro-magnetic stimulation of the vagus nerve. Front

Physiol. 2022;13:938101.

Ji N, Lin WH, Chen F, Xu L, Huang J, Li G. Blood pressure modulation with low-intensity focused

ultrasound stimulation to the vagus nerve: a pilot animal study. Front Neurosci. 2020;14:586424.

Jones P, Guillaud L, Desbois C, Benoist JF, Combrisson H, Dauger S, et al. Pathology influences blood

pressure change following vagal stimulation in an animal intubation model. PLoS One. 2013;8(8):e69957.

Kalarus Z, Merkely B, Neužil P, Grabowski M, Mitkowski P, Marinskis G, et al. Pacemaker-Based

Cardiac Neuromodulation Therapy in Patients With Hypertension: A Pilot Study. J Am Heart Assoc. 2021;10(16):e020492.

Kansal N, Clair DG, Jaye DA, Scheiner A. Carotid baroreceptor stimulation blood pressure response

mapped in patients undergoing carotid endarterectomy (C-Map study). Autonomic Neuroscience. 2016;201:60–7.

Kawabe T, Kawabe K, Sapru HN. Cardiovascular responses to somatosensory stimulation and their

modulation by baroreflex mechanisms. Clin Exp Hypertens. 2007;29(6):403–18.

Kerman IA, Emanuel BA, Yates BJ. Vestibular stimulation leads to distinct hemodynamic patterning.

American Journal of Physiology-Regulatory, Integrative and Comparative Physiology. 2000;279(1):R118–R125.

Kim HK, Hotta N, Ishizawa R, Iwamoto GA, Vongpatanasin W, Mitchell JH, et al. Exaggerated pressor

and sympathetic responses to stimulation of the mesencephalic locomotor region and exercise pressor reflex in type 2 diabetic rats. American Journal of Physiology-Regulatory, Integrative and Comparative Physiology. 2019;317(2):R270–R279.

Koeda S, Ishii H, Kuchiiwa S, Izumi H. Role of the spinal trigeminal nucleus in the rat autonomic reflex.

Arch Oral Biol. 2009;54(12):1136–42.

Lee HJ, White JM, Chung J, Malone P, DeWeerth SP, Tansey KE. Differential cardiovascular responses

to cutaneous afferent subtypes in a nociceptive intersegmental spinal reflex. Sci Rep. 2019;9(1):19049.

Li D, Cao F, Han J, Wang M, Lai C, Zhang J, et al. The sustainable antihypertensive and target organ

damage protective effect of transcranial focused ultrasound stimulation in spontaneously hypertensive rats. J Hypertens. 2023;41(5):852–66.

Li D, Cui Z, Xu S, Xu T, Wu S, Bouakaz A, et al. Low-intensity focused ultrasound stimulation treatment

decreases blood pressure in spontaneously hypertensive rats. IEEE Trans Biomed Eng. 2020;67(11):3048–56.

Li M, Tjen-A-Looi SC, Guo ZL, Longhurst JC. Electroacupuncture modulation of reflex hypertension

in rats: role of cholecystokinin octapeptide. American Journal of Physiology-Regulatory, Integrative and Comparative Physiology. 2013;305(4):R404–R413.

Li M, Tjen-A-Looi SC, Guo ZL, Longhurst JC. Repetitive electroacupuncture attenuates cold-induced

hypertension through enkephalin in the rostral ventral lateral medulla. Sci Rep. 2016;6(1):35791.

Liang N, Iwamoto GA, Downey RM, Mitchell JH, Smith SA, Mizuno M. The pressor response to

concurrent stimulation of the mesencephalic locomotor region and peripheral sensory afferents is attenuated in normotensive but not hypertensive rats. Front Physiol. 2019;10:95.

Liang N, Mitchell JH, Smith SA, Mizuno M. Exaggerated sympathetic and cardiovascular responses to

stimulation of the mesencephalic locomotor region in spontaneously hypertensive rats. Am J Physiol Heart Circ Physiol. 2016;310(1):H123-31.

Linz D, Mahfoud F, Schotten U, Ukena C, NEUBERGER HR, Wirth K, et al. Effects of electrical

stimulation of carotid baroreflex and renal denervation on atrial electrophysiology. J Cardiovasc Electrophysiol. 2013;24(9):1028–33.

Liu HK, Guild SJ, Ringwood J V, Barrett CJ, Leonard BL, Nguang SK, et al. Dynamic baroreflex control

of blood pressure: influence of the heart vs. peripheral resistance. American Journal of Physiology-Regulatory, Integrative and Comparative Physiology. 2002;283(2):R533–R542.

Lohmeier TE, Iliescu R, Dwyer TM, Irwin ED, Cates AW, Rossing MA. Sustained suppression of

sympathetic activity and arterial pressure during chronic activation of the carotid baroreflex. American Journal of Physiology-Heart and Circulatory Physiology. 2010;299(2):H402–H409.

López-González MV, D\’\iaz-Casares A, González-Garc\’\ia M, Peinado-Aragonés CA, Barbancho MA,

de Albornoz M, et al. Glutamate receptors of the A5 region modulate cardiovascular responses evoked from the dorsomedial hypothalamic nucleus and perifornical area. J Physiol Biochem. 2018;74:325–34.

Madhavan M, Desimone C V, Ebrille E, Mulpuru SK, Mikell SB, Johnson SB, et al. Transvenous

stimulation of the renal sympathetic nerves increases systemic blood pressure: a potential new treatment option for neurocardiogenic syncope. J Cardiovasc Electrophysiol. 2014;25(10):1115–8.

Matsukawa K, Ishii K, Ishida T, Nagai A, Liang N. Stimulation of the mesencephalic ventral tegmental

area blunts the sensitivity of cardiac baroreflex in decerebrate cats. Autonomic Neuroscience. 2014;189:16–24.

Matsukawa K, Nakamoto T, Liang N. Electrical stimulation of the mesencephalic ventral tegmental area

evokes skeletal muscle vasodilatation in the cat and rat. The Journal of Physiological Sciences. 2011;61:293–301.

Moreira BR, Duque AP, Massolar CS, R de LP, Mediano MFF, Guimarães TCF, et al. Transcutaneous

Electrical Stimulation of PC5 and PC6 Acupoints Modulates Autonomic Balance in Heart Transplant Patients: A Pilot Study. J Acupunct Meridian Stud. 2019;12(3):84–9.

Mun J, Lee J, Park E, Park SM. Frequency-dependent depression of the NTS synapse affects the

temporal response of the antihypertensive effect of auricular vagus nerve stimulation (aVNS). J Neural Eng. 2022;19(4):46039.

Mun J, Lee J, Park SM. Real-time closed-loop brainstem stimulation modality for enhancing temporal

blood pressure reduction. Brain Stimul. 2024;17(4):826–35.

Nakahara H, Kawada T, Ueda SY, Kawai E, Yamamoto H, Sugimachi M, et al. Electroacupuncture most

effectively elicits depressor and bradycardic responses at 1 Hz in humans. Clin Auton Res. 2016;26(1):59–66.

O’Callaghan EL, McBryde FD, Patel NK, Paton JFR. Examination of the periaqueductal gray as a site

for controlling arterial pressure in the conscious spontaneously hypertensive rat. Autonomic Neuroscience. 2022;240:102984.

Ohshita N, Nakajo N, Takemura M. Characteristics of the trigeminal depressor response in cats. J

Neurosci Res. 2004;76(6):891–901.

Ong J, Kinsman BJ, Sved AF, Rush BM, Tan RJ, Carattino MD, et al. Renal sensory nerves increase

sympathetic nerve activity and blood pressure in 2-kidney 1-clip hypertensive mice. J Neurophysiol. 2019;122(1):358–67.

Patel S, Krishna V, Nicholas J, Welzig CM, Vera C. Preliminary observations on the vasomotor responses

to electrical stimulation of the ventrolateral surface of the human medulla. J Neurosurg. 2012;117(1):150–5.

Phillips AA, Squair JW, Sayenko DG, Edgerton VR, Gerasimenko Y, Krassioukov A V. An autonomic

neuroprosthesis: noninvasive electrical spinal cord stimulation restores autonomic cardiovascular function in individuals with spinal cord injury. J Neurotrauma. 2018;35(3):446–51.

Plachta DTT, Gierthmuehlen M, Cota O, Espinosa N, Boeser F, Herrera TC, et al. Blood pressure control

with selective vagal nerve stimulation and minimal side effects. J Neural Eng. 2014;11(3):36011.

Plachta DTT, Zentner J, Aguirre D, Cota O, Stieglitz T, Gierthmuehlen M. Effect of cardiac-cycle-

synchronized selective vagal stimulation on heart rate and blood pressure in rats. Adv Ther. 2016;33:1246–61.

Ramadhani R, Sato T, Okada Y, Ohke H, Ishii H. Differences in the regulatory mechanism of blood flow

in the orofacial area mediated by neural and humoral systems. Journal of Comparative Physiology B. 2023;193(1):109–24.

Rodrigues B, Barboza CA, Moura EG, Ministro G, Ferreira-Melo SE, Castano JB, et al. Acute and short-

term autonomic and hemodynamic responses to transcranial direct current stimulation in patients with resistant hypertension. Front Cardiovasc Med. 2022;9:853427.

Sachdeva R, Nightingale TE, Pawar K, Kalimullina T, Mesa A, Marwaha A, et al. Noninvasive

neuroprosthesis promotes cardiovascular recovery after spinal cord injury. Neurotherapeutics. 2021;18(2):1244–56.

Saleh TM, Saleh MC, Connell BJ. Estrogen blocks the cardiovascular and autonomic changes following

vagal stimulation in ovariectomized rats. Autonomic Neuroscience. 2001;88(1–2):25–35.

Salman IM, Ameer OZ, McMurray S, Hassan SF, Sridhar A, Lewis SJ, et al. Low intensity stimulation

of aortic baroreceptor afferent fibers as a potential therapeutic alternative for hypertension treatment. Sci Rep. 2022;12(1):12242.

Sanchez-Larsen A, Principe A, Ley M, Vaquerizo B, Langohr K, Rocamora R. Insular role in blood

pressure and systemic vascular resistance regulation. Neuromodulation: Technology at the Neural Interface. 2024;27(7):1218-26.

Santarnecchi E, Feurra M, Barneschi F, Acampa M, Bianco G, Cioncoloni D, et al. Time course of

corticospinal excitability and autonomic function interplay during and following monopolar tDCS. Front Psychiatry. 2014;5:86.

Schmidli J, Savolainen H, Eckstein F, Irwin E, Peters TK, Martin R, et al. Acute device-based blood

pressure reduction: electrical activation of the carotid baroreflex in patients undergoing elective carotid surgery. Vascular. 2007;15(2):63–9.

Schultz DM, Musley S, Beltrand P, Christensen J, Euler D, Warman E. Acute cardiovascular effects of

epidural spinal cord stimulation. Pain Physician. 2007;10(5):677.

Sesa-Ashton G, Wong R, McCarthy B, Datta S, Henderson LA, Dawood T, et al. Stimulation of the

dorsolateral prefrontal cortex modulates muscle sympathetic nerve activity and blood pressure in humans. Cereb Cortex Commun. 2022;3(2):tgac017.

Shah J V, Collar BJ, Ditslear E, Irazoqui PP. An ASIC system for closed-loop blood pressure modulation

through right cervical vagus nerve stimulation. IEEE Trans Biomed Eng. 2022;69(10):3021–8.

Šinkovec M, Trobec R, Kamenski T, Jerman N, Meglič B. Hemodynamic responses to low-level

transcutaneous auricular nerve stimulation in young volunteers. IBRO Neurosci Rep. 2023;14:154–9.

Šinkovec M, Trobec R, Meglic B. Cardiovascular responses to low-level transcutaneous vagus nerve

stimulation. Autonomic Neuroscience. 2021;236:102851.

Solinsky R, Burns K, Tuthill C, Hamner JW, Taylor JA. Transcutaneous spinal cord stimulation and its

impact on cardiovascular autonomic regulation after spinal cord injury. American Journal of Physiology-Heart and Circulatory Physiology. 2024;326(1):H116–H122.

Stauss HM. Differential hemodynamic and respiratory responses to right and left cervical vagal nerve

stimulation in rats. Physiol Rep. 2017;5(7):e13244.

Sun J, Scherlag BJ, He BO, Shen X, Gao M, Zhang L, et al. Electrical stimulation of vascular autonomic

nerves: effects on heart rate, blood pressure, and arrhythmias. Pacing and Clinical Electrophysiology. 2015;38(7):825–30.

Sun X, Lan Q qian, Cai Y, Yu Y qin. Electrical stimulation of deep peroneal nerve mimicking

acupuncture inhibits the pressor response via capsaicin-insensitive afferents in anesthetized rats. Chin J Integr Med. 2012;18(2):130–6.

Suzuki A, Shimura M. Changes in blood pressure induced by electrical stimulation of the femur in

anesthetized rats. Auton Neurosci. 2010;158(1–2):39–43.

Sverrisdóttir YB, Green AL, Aziz TZ, Bahuri NFA, Hyam J, Basnayake SD, et al. Differentiated

baroreflex modulation of sympathetic nerve activity during deep brain stimulation in humans. Hypertension. 2014;63(5):1000–10.

Sverrisdottir YB, Martin SC, Hadjipavlou G, Kent AR, Paterson DJ, FitzGerald JJ, et al. Human dorsal

root ganglion stimulation reduces sympathetic outflow and long-term blood pressure. Basic to Translational Science. 2020;5(10):973–85.

Tanaka S, Masuda T, Kamiya K, Hamazaki N, Akiyama A, Kamada Y, et al. A Single Session of

Neuromuscular Electrical Stimulation Enhances Vascular Endothelial Function and Peripheral Blood Circulation in Patients With Acute Myocardial Infarction A Quasi-Randomized Controlled Trial. Int Heart J. 2016;57(6):676–81.

Tarasova O, Borovik A, Tsvirkoun D, Lebedev V, Steeves J, Krassioukov A. Orthostatic response in rats

after hindlimb unloading: effect of transcranial electrical stimulation. Aviat Space Environ Med. 2007;78(11):1023–8.

Thornton JM, Aziz T, Schlugman D, Paterson DJ. Electrical stimulation of the midbrain increases heart

rate and arterial blood pressure in awake humans. J Physiol. 2002;539(2):615–21.

Turner MJ, Kawada T, Shimizu S, Sugimachi M. Sustained reduction in blood pressure from electrical

activation of the baroreflex is mediated via the central pathway of unmyelinated baroreceptors. Life Sci. 2014;106(1–2):40–9.

Voustianiouk A, Kaufmann H, Diedrich A, Raphan T, Biaggioni I, MacDougall H, et al. Electrical

activation of the human vestibulo-sympathetic reflex. Exp Brain Res. 2006;171:251–61.

Wallbach M, Halbach M, Reuter H, Passauer J, Lüders S, Böhning E, et al. Baroreflex activation therapy

in patients with prior renal denervation. J Hypertens. 2016;34(8):1630–8.

Wang J, Chen J, Shu L, Zhang R, Dai M, Fang X, et al. Carotid baroreceptor stimulation improves

pulmonary arterial remodeling and right ventricular dysfunction in pulmonary arterial hypertension. Basic to Translational Science. 2024;9(4):475–92.

Watanabe H, Ishii H, Niioka T, Yamamuro M, Izumi H. Occurrence of parasympathetic vasodilator fibers

in the lower lip of the guinea-pig. Journal of Comparative Physiology B. 2008;178:297–305.

Yang B, Wang Y, Zhang F, Ju W, Chen H, Mika Y, et al. Rationale and evidence for the development of

a durable device-based cardiac neuromodulation therapy for hypertension. Journal of the American Society of Hypertension. 2018;12(5):381–91.

Yasuda M, Izumi H. Trigeminal nerve-mediated reflex arterial blood pressure decrease and

vasodilatation in lower lip of the rabbit. Brain Res. 2003;987(1):59–66.

Yoshida T, Masani K, Sayenko DG, Miyatani M, Fisher JA, Popovic MR. Cardiovascular response of

individuals with spinal cord injury to dynamic functional electrical stimulation under orthostatic stress. IEEE Trans Neural Syst Rehabil Eng. 2012;21(1):37–46.

Yu L, Huang B, Wang Z, Wang S, Wang M, Li X, et al. Impacts of renal sympathetic activation on atrial

fibrillation: the potential role of the autonomic cross talk between kidney and heart. J Am Heart Assoc. 2017;6(3):e004716.

Zafeiropoulos S, Ahmed U, Bekiaridou A, Jayaprakash N, Mughrabi IT, Saleknezhad N, et al.

Ultrasound neuromodulation of an anti-inflammatory pathway at the spleen improves experimental pulmonary hypertension. Circ Res. 2024;135(1):41–56.

Zhang J, Cao Q, Li R, Hu J, Dai J, Zhang H, et al. Hemodynamic responses to magnetic stimulation of

carotid sinus in normotensive rabbits. J Hypertens. 2017;35(8):1676–84.

Zheng M, Deng KQ, Wang X, Luo D, Qu W, Chen C, et al. Pulmonary Artery Denervation Inhibits Left Stellate Ganglion Stimulation-Induced Ventricular Arrhythmias Originating From the RVOT. JACC Clin Electrophysiol. 2023;9(8 Pt 1):1354–67.
